# A comprehensive review and evaluation of species richness estimation

**DOI:** 10.1093/bib/bbaf158

**Published:** 2025-04-11

**Authors:** Johanna Elena Schmitz, Sven Rahmann

**Affiliations:** Algorithmic Bioinformatics, Center for Bioinformatics Saar, Saarland Informatics Campus, 66123 Saarbrücken, Germany; Fakultät MI, Saarland University, Saarland Informatics Campus, 66123 Saarbrücken, Germany; Saarbrücken Graduate School of Computer Science, Saarland Informatics Campus, 66123 Saarbrücken, Germany; Algorithmic Bioinformatics, Center for Bioinformatics Saar, Saarland Informatics Campus, 66123 Saarbrücken, Germany; Fakultät MI, Saarland University, Saarland Informatics Campus, 66123 Saarbrücken, Germany

**Keywords:** species richness, diversity estimation, upsampling, immune repertoire, microbiome, comparative evaluation

## Abstract

**Motivation:**

The statistical problem of estimating the total number of distinct species in a population (or distinct elements in a multiset), given only a small sample, occurs in various areas, ranging from the unseen species problem in ecology to estimating the diversity of immune repertoires. Accurately estimating the true richness from very small samples is challenging, in particular for highly diverse populations with many rare species. Depending on the application, different estimation strategies have been proposed that incorporate explicit or implicit assumptions about either the species distribution or about the sampling process. These methods are scattered across the literature, and an extensive overview of their assumptions, methodology, and performance is currently lacking.

**Results:**

We comprehensively review and evaluate a variety of existing methods on real and simulated data with different compositions of rare and abundant species. Our evaluation shows that, depending on species composition, different methods provide the most accurate richness estimates. Simple methods based on the observed number of singletons yield accurate asymptotic lower bounds for several of the tested simulated species compositions, but tend to underestimate the true richness for heterogeneous populations and small samples containing 1% to 5% of the population. When the population size is known, upsampling (extrapolating) estimators such as PreSeq and RichnEst yield accurate estimates of the total species richness in a sample that is up to 10 times larger than the observed sample.

**Availability:**

Source code for data simulation and richness estimation is available at https://gitlab.com/rahmannlab/speciesrichness.

## Introduction

Estimating the diversity of a population from a small sample has a wide range of applications in diverse fields, such as ecology, immunology, biological sequence analysis, and linguistics. One of the oldest applications is the unseen species problem in ecology, e.g. predicting the number of butterfly species on an island after capturing a small collection of butterflies [1]. The same statistical problem arises in linguistics when trying to estimate how many words a writer might have known but never used in any of his published works. In quantitative linguistics, this is a measure to compare the vocabulary richness of writers [2]. Recent applications include the analysis of microbial complexity in environmental niches [3], the comparison of bacterial diversity in human guts under different disease conditions [4], or the quantification of a suitable sequencing depth to study rare cancer types based on the diversity of genetic variants and mutations [5].

While there are many measures of diversity, such as the proportion of rare and abundant species, or the entropy of the species distribution, we limit this review to the estimation of *species richness* from individual-based abundance data. Species richness measures the total number of distinct species in a population, assuming that each individual belongs to a single species. We hence exclude methods that measure presence and absence of a species in a sampling unit (called incidence data), require spatial information, or assume that an individual may belong to several species at once.

In most applications, it is infeasible to observe the complete population; so, the *observed* species richness of a sample usually underestimates the *true* richness, especially for populations with many rare species. However, an accurate estimate is crucial to analyse the properties of a population. For instance, the T- or B-cell receptor richness of immune repertoires indicates the effectiveness of the immune system, and an accurate estimate is thus vital to compare immune systems between healthy and diseased individuals. Since the frequency distribution of T-cell receptor repertoires is highly skewed, rare T-cell receptors are often missed in the sampling process [6]. This necessitates robust estimation of the actual richness.

**Figure 1:**
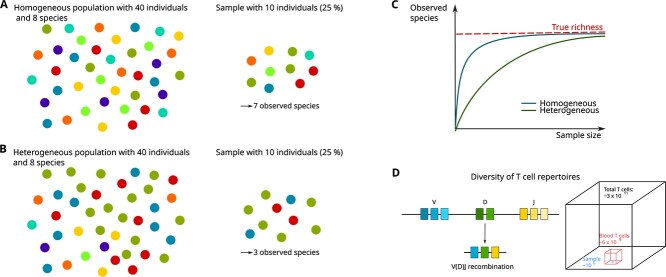
Comparison of species (color) richness in the population and in a sample. **A** For a homogeneous population, a small sample is sufficient to observe a sample richness that is close to the true richness. **B** The sample richness of a population with many rare species underestimates the true richness. **C** Rarefaction curve. Increasing the sample size leads to an increase in the observed richness, converging to the true richness. For a heterogeneous population with many rare species, the convergence is slow. **D** T-cell diversity. Somatic recombination of V, D, J gene segments during T-cell maturation gives rise to numerous distinct T-cell receptors that form an immune system that is able to recognize almost all potential pathogens. However, analysing T-cell receptor diversity based on a small blood sample is challenging, because the sample only contains a minute portion of all T-cells from a person.

Accurate estimation of species richness is a challenging statistical problem, in particular without making additional assumptions about the sampling process or the species distribution (see Fig. 1). Various estimators have been proposed over the years to achieve accurate richness estimates for populations with different species compositions. Early estimators, like the Chao 1 [7] or Jackknife estimator [8], assume that most information about the number of missing species is present in the number of species captured only once or twice. Other estimators assume that the species counts follow a parametric probability distribution, e.g. a Gamma-Poisson mixture distribution [9]. Several recent methods make no such assumptions and are based on linear programming [10, 11] or curve fitting [6, 12].

While for ecological studies one is often interested in either accurate asymptotic lower bounds of the population size or species richness estimates for extrapolated sample sizes up to $2$ to $3$ times the observed sample size [13], other applications, such as metagenomics or immune repertoire analysis, require accurate estimates for populations that are more than 10 times larger than the observed sample. Hence, new methods are still being developed with the goal to yield reliable results for populations that are several magnitudes larger than the observed sample [14].

In addition, data from various research areas have different properties that may invalidate the assumption of some richness estimators. For example, in microbiome analyses, methods relying on accurate abundances of rare species may lead to over- or underestimation of species richness depending on the performed preprocessing. When the species richness is decided on the sequence level, singletons are likely caused by sequencing errors and are often removed prior to further analysis steps. When we estimate species richness at the taxonomic level, contamination or misclassification may result in some species being incorrectly present or absent [15]. Such application specific problems should be considered before applying species richness estimators; we come back to these important points in the Discussion.

Since a systematic comparison of long-established and contemporary species richness estimators has not yet been conducted, we evaluate the performance of species richness estimators on a variety of simulated and real data with different underlying frequency distributions.

## Methods

### Definitions and notation

From a full population of $N$ individuals ($N$ may be finite or infinite, known or unknown), a finite random sample of $n$ individuals is observed. We assume that the sample is small, i.e. $n \ll N$. Each individual (sometimes called element) in the population belongs to a species (sometimes called class or group). The number of species in the full population is referred to as its *species richness*  $S$, which we assume to be finite (even for infinite $N$).

The *observed sample richness* is denoted by $S_{\text{obs}}$.

For each observed species, we count its abundance in the sample and obtain the *abundance* vector $a = (a_{i})_{1 \leq i \leq S_{\text{obs}}}$.

The number of species observed exactly $k$ times in the sample is given by $f_{k}$ (i.e. $f_{k} = | \{i \,|\, a_{i} = k,\, 1 \leq i \leq S_{\text{obs}} \} | $), such that the number $n$ of individuals in the sample satisfies 


\begin{align*} n = \sum_{i=1}^{S_{\text{obs}}} a_{i} = \sum_{k \ge 1}\, k \, f_{k} \,, \end{align*}

and the observed richness is given by 


\begin{align*} S_{\text{obs}} = \sum_{k \ge 1}\, f_{k} \,. \end{align*}

The population’s species richness can be expressed by 


\begin{align*} S = \sum_{k \ge 0}\, f_{k} \;=\; S_{\text{obs}} + f_{0} \,, \end{align*}

i.e. the observed richness plus the number of unobserved species that are missing in the sample. Therefore, estimating $S$ is equivalent to estimating the unobserved $f_{0}$ from the observed $f = (f_{1}, f_{2}, \dots )$. As the observation is finite, $f$ is of finite length.

### Classification of richness estimators

Species richness estimators can be divided into two main groups: (1) If the total population size $N$ is known or assumed to be known, finite, and given as an input, we have an *upsampling* or *extrapolation* task (by a factor of $N/n$), i.e. we need to solve the inverse problem of the random (down)sampling process. (2) If $N$ is unknown or assumed infinite, it is (often implicitly) assumed that the total population species richness $S$ is finite, i.e. the rarefaction curve reaches a finite asymptotic upper limit (Fig. 1C), which means that there cannot be arbitrarily many rare species. The first group is referred to as *upsampling estimators* and the second group as *population estimators*. In ecology, the two groups are often called *extrapolating* and *asymptotic* richness estimators, respectively. If (an approximation of) $N$ is available, an upsampling estimator should be preferred, as using more information typically yields more accurate results.

#### Scenarios for upsampling (extrapolating) and population (asymptotic) estimators

A use case for an upsampling estimator is the decision on the sequencing depth of a DNA library of unknown quality. Based on PCR duplicate statistics after a low-depth (say, 3x coverage) pre-experiment, it can be decided whether 30x versus 15x coverage would yield significantly more new fragments, or whether one would see mostly PCR duplicates. A use case for both classes is the estimation of T-cell receptor richness from small blood samples (e.g. in healthy versus sick individuals): The exact total number of T-cells is unknown, but we have reasonable estimates of the total number of T-cells in the body and in the blood (Fig. 1D). A use case for population estimators is the estimation of microbiome species diversity of the intestine, bacterial species diversity in soil or global insect, plant, or animal diversity in conservation studies.

#### Classification of estimators by assumptions made

Estimators can be further divided according to whether they make assumptions about the species composition, i.e. about the behavior of $(f_{k})_{k\ge 0}$. If they do, the species richness estimation problem often simplifies to estimating one or a few parameters of a parametric distribution, which leads to computationally efficient estimators that show good accuracy if the assumptions are satisfied, but that may be inaccurate if not. We call these estimators *parametric estimators*, and estimators that make no explicit distributional assumptions *non-parametric estimators*.

An overview of estimators that we discuss in more detail in the following sections appears in Table 1. Each particular method may make explicit or implicit additional assumptions, which we shall describe below as needed. We first mention several common principles behind these methods.

**Table 1:** Overview of species richness estimators. Column “up” indicates whether an estimator is an upsampling estimator (${\color{darkgreen}{\checkmark }}$) or not (${\color{darkred}{\times }}$; then it is a population estimator). Population (asymptotic) and upsampling (extrapolating) estimators are separated by a horizontal line. Column “par” indicates whether an estimator is parametric (${\color{darkgreen}{\checkmark }}$), i.e. whether it makes distributional assumptions about the species composition, or not (${\color{darkred}{\times }}$). ${}^{*}$The Chao 1 estimator can be derived from a Poisson model, but was first introduced as a nonparametric estimator. Column “Implementation” links to the estimator’s implementation that we used for computational experiments (“ours” means our own implementation; available at https://gitlab.com/rahmannlab/speciesrichness).

Name	Up	Par	Reference	Implementation
Good–Turing	${\color{darkred}{\times }}$	${\color{darkred}{\times }}$	Good [2]	ours
Jackknife	${\color{darkred}{\times }}$	${\color{darkred}{\times }}$	Burnham and Overton [8]	ours
ACE	${\color{darkred}{\times }}$	${\color{darkred}{\times }}$	Chao and Lee Chao and Lee [16]	ours
Poisson	${\color{darkred}{\times }}$	${\color{darkgreen}{\checkmark }}$	Sandland and Cormack [9]	Breakaway R package
Chao 1	${\color{darkred}{\times }}$	${\color{darkred}{\times }}$ ${\color{darkgreen}{\checkmark }}^{*}$	Chao [7]	ours
Gamma-Poisson mixture	${\color{darkred}{\times }}$	${\color{darkgreen}{\checkmark }}$	Fisher *et al.* [1]	ours
Chao and Bunge	${\color{darkred}{\times }}$	${\color{darkgreen}{\checkmark }}$	Chao and Bunge [17]	Breakaway R package
Lanumteang and Böhning	${\color{darkred}{\times }}$	${\color{darkgreen}{\checkmark }}$	Lanumteang and Böhning [18]	ours
Chiu	${\color{darkred}{\times }}$	${\color{darkgreen}{\checkmark }}$	Chiu [19]	ours
Objective Bayesian	${\color{darkred}{\times }}$	${\color{darkgreen}{\checkmark }}$	Barger and Bunge [20]	Breakaway R package
Recon	${\color{darkred}{\times }}$	${\color{darkred}{\times }}$	Kaplinsky and Arnaout [14]	GitHub ArnaoutLab
Valiant	${\color{darkred}{\times }}$	${\color{darkred}{\times }}$	Valiant and Valiant [11]	Valiant Code
Breakaway	${\color{darkred}{\times }}$	${\color{darkred}{\times }}$	Willis and Bunge [12]	Breakaway R package
TES	${\color{darkred}{\times }}$	${\color{darkgreen}{\checkmark }}$	Zou *et al.* [21]	TES R script
iNEXT	${\color{darkgreen}{\checkmark }}$	${\color{darkred}{\times }}$	Hsieh *et al.* [22]	iNEXT R package
Smoothed Good–Toulmin	${\color{darkgreen}{\checkmark }}$	${\color{darkgreen}{\checkmark }}$	Orlitsky *et al.* [23]	ours
PreSeq	${\color{darkgreen}{\checkmark }}$	${\color{darkred}{\times }}$	Daley and Smith [24]	PreSeq R package
Pitman sampling formula	${\color{darkgreen}{\checkmark }}$	${\color{darkgreen}{\checkmark }}$	Pitman [25]	GitHub Stefanie Tauber
DivE	${\color{darkgreen}{\checkmark }}$	${\color{darkred}{\times }}$	Laydon *et al.* [26]	DivE R package
RichnEst (formerly Dupre)	${\color{darkgreen}{\checkmark }}$	${\color{darkred}{\times }}$	Schröder and Rahmann [10]	GitLab RahmannLab

### General principles

Given the abundance vector $a=(a_{i})$, $i=1,\dots ,S_{\text{obs}}$ of the observed species in a sample, we assume that it is the realization of an underlying probabilistic model. Let $X$ denote the random variable describing the abundance of a randomly picked species; let $p_{k}$ be the probability of observing a species exactly $k$ times; so ${\mathbb{P}}[X=k] = p_{k}$ for $k=0,1,2,\dots $. If we draw $S$ times an independent copy of $X$ (abundances, including zeros) and count how many times each abundance $k$ was seen, we obtain $f=(f_{k})$, including $f_{0}$. Conversely, $f_{k}/S$ is an estimate for $p_{k}$.

#### Zero-truncated distribution

The number of unobserved species $f_{0}$ is unknown. Thus, if $P = (p_{k})_{k\ge 0}$ with $p_{k}$ being the true probability distribution for capturing a species exactly $k$ times (whether it follows a parametric family or not), then $P^{+} = (p^{+}_{k})_{k \ge 1}$ with $p^{+}_{k} = p_{k} / (1-p_{0})$ for $k\ge 1$ is the *zero-truncated* distribution. It is obtained from $P$ by setting $p^{+}_{0}:= 0$ and $p^{+}_{k} = p_{k}/Z$ for $k\ge 1$, where the normalization constant $Z$ ensures that $\sum _{k\ge 1}\, p^{+}_{k} = 1$, so $Z = 1 - p_{0} = S_{\text{obs}}/S$. From this, we derive 


(1) \begin{align*} & S = S_{\text{obs}} / (1-p_{0}) \,,\end{align*}

which by itself is not very helpful, as both $S$ and $p_{0}$ are unknown, but with additional (distributional) assumptions yields useful estimators (see below). Obtaining $S$ from equation (1) is also referred to as the Horvitz–Thompson point estimate for zero-truncated distributions [27].

#### Coverage estimates

The denominator in equation (1), $1-p_{0}$ or $1-{\mathbb{P}}[X=0] = {\mathbb{P}}[X\ge 1]$, reflects the proportion of observed species and is also called sample *coverage*  $C$. Some of the estimators directly or indirectly estimate $C$ as $\hat{C}$ and then $\hat{S} = S_{\text{obs}} / \hat{C}$.

#### Distributional assumption: poisson

As mentioned above, one may assume that $a=(a_{i})_{1 \leq i \leq S_{\text{obs}}}$ follows a certain type of probability distribution with probabilities $p_{k} \approx f_{k}/S$ for $k\ge 0$. Such an assumption cannot be justified in general, but may hold for certain types of datasets, and it simplifies the estimation problem. A popular distributional assumption for each $a_{i}$ is the Poisson distribution, which models the random number $X$ of successes when many attempts ($n\to \infty $) are made, each with a very small success probability ($p\to 0$), such that their product $\lambda := pn> 0$, corresponding to the expected number of successes, is a positive constant. Then, the Poisson distribution specifies that $p_{k} = {\mathbb{P}}[X=k]=\text{e}^{-\lambda } \cdot \lambda ^{k} / k!$. The Poisson assumption can be exploited in different ways.

First, the Poisson distribution specifies that $p_{0} = p_{1} = \text{e}^{-\lambda }$, so we can assume that $f_{0}\approx f_{1}$ and simply estimate $\hat{S} = S_{\text{obs}} + f_{1}$. This estimator can also be derived in a non-parametric way as a Jackknife estimator (see below).

Alternatively, under the Poisson assumption, ${\mathbb{P}}[X=1] / {\mathbb{P}}[X=0] = \lambda $ can be estimated by $f_{1} / f_{0}$, and ${\mathbb{P}}[X=2]/{\mathbb{P}}[X=1] = \lambda /2$ can be estimated by $f_{2}/ f_{1}$. It follows that $f_{1}/f_{0} \approx 2\ {}f_{2}/f_{1}$, or $f_{0} \approx f_{1}^{2}/(2\, f_{2})$, which is essentially the Chao 1 estimator (see the following section). Note that this estimator only uses $f_{1}$ and $f_{2}$ and not the other information contained in the data.

Still under the Poisson assumption, the data can be more comprehensively used if we compute an maximum likelihood estimate for the parameter $\lambda $ from the observed zero-truncated Poisson distribution and then use equation (1) to estimate $\hat{S} = S_{\text{obs}} / (1 - \text{e}^{-\lambda })$. This is the Poisson (PO) estimator (details in the following section).

### Estimators in detail

#### Population estimators

##### Good–Turing estimator (GT)

The Good–Turing estimator is one of the earliest richness estimators. Assuming that a *random* sample is drawn from an *infinite* population with a *finite* number of species $S$, Good [2] proposed estimates for the probabilities that a species is represented exactly $r$ times without making further assumptions about the population frequency distribution. One of their main results is that the proportion of species represented in the sample (coverage) is approximately $\hat{C} = 1 - f_{1}/n$, or equivalently, the probability that the next observed individual belongs to an unseen species is given by $\hat{p}_{0} = f_{1}/n$. This result led to the common assumption that rare species, especially the number of singletons, contain most information about the number of missing species.

Good [2] did not further comment on predicting species richness given the probability to observe a new element. However, we may use the estimate $\hat{p}_{0} = f_{1}/n$ (or $\hat{C} = 1 - f_{1}/n$) together with equation (1) or the coverage estimate to obtain the GT estimator 


\begin{align*} \hat{S}_{\text{GT}} &= \frac{S_{\text{obs}}}{1 - f_{1}/n} = S_{\text{obs}} / \hat{C} \;. \end{align*}

##### Jackknife estimators (Jack 1, Jack 2)

Burnham and Overton [8] derived *non-parametric* estimators that are a linear combination of the species frequencies. The derivation is based on the following assumptions: the population is *closed*, the species detection rate is *constant* for each species but may vary between species and the capture events are all *independent*. It follows that the observed capture frequencies are a *random* variable following a *multinomial* distribution with unknown success probabilities [28]. Instead of assuming a parametric distribution for the success probabilities, Burnham and Overton [8] derive a non-parametric estimator using the generalized Jackknife method. The first and second order Jackknife estimators are given by 


\begin{align*} \hat{S}_{\text{Jack 1}} &= S_{\text{obs}} + f_{1} \,, \\ \hat{S}_{\text{Jack 2}} &= S_{\text{obs}} + 2f_{1} - f_{2} \,.\\ \end{align*}

#### ACE estimator (ACE)

The abundance-based coverage estimator (ACE) is a modification of the GT estimator in the sense that it considers only *rare* species for the coverage estimator. The species are separated into rare and abundant groups based on a frequency cutoff $T$, i.e. species are rare if they are observed at most $T$ times [29]. The most common cutoff is $T = 10$, but results are sensitive to the choice of $T$.

We apply $\hat{S}_{\text{GT}}$, but instead of using the entire number $S_{\text{obs}}$ of observed species, we only use the number $S_{\text{rare}}$ of rare species and adjust $n$ accordingly. The number of abundant species is simply counted as-is. We obtain 


\begin{align*} & \hat{S} = S_{\text{abund}} + \frac{S_{\text{rare}}}{\hat{C}_{\text{rare}}}\,, \end{align*}

where 


\begin{align*} & S_{\text{abund}} = \sum_{k=T+1}^{\infty}\, f_{k} \,, \qquad S_{\text{rare}} = \sum_{k=1}^{T}\, f_{k}\,, \end{align*}

\begin{equation*} n_{\text{rare}} = \sum_{k=1}^{T}\, k\, f_{k} \,, \qquad \hat{C}_{\text{rare}} = 1 - \frac{f_{1}}{n_{\text{rare}}} \,. \end{equation*}

The above estimator is not the final ACE estimator because it assumes that all rare species are *homogeneous*, i.e. all species are assumed to have the same relative abundance. Since the homogeneity assumption may be violated, Chao and Lee [16] proposed an adjusted estimator that accounts for the *heterogeneity* of rare elements. For a population with true relative species abundances $(q_{i})_{1 \leq i \leq S}$, the abundance distribution may be summarized by its mean $\bar{q} = 1 / S$ and coefficient of variation (CV), where the squared CV is defined as 


\begin{align*} & \gamma^{2} = \frac{1}{S} \sum_{i=1}^{S} \frac{(q_{i} - \bar{q})^{2}}{\bar{q}^{2}} \;. \end{align*}

Based on the results by Good and Toulmin [30], Chao and Lee [16] estimate $\gamma ^{2}$ by 


\begin{align*} & \hat{\gamma}^{2} = \max \left\{ 0,\; \frac{S_{\text{rare}}}{C_{\text{rare}}} \frac{\sum_{k=1}^{T}\, k \, (k-1) \, f_{k}}{n_{\text{rare}} \, (n_{\text{rare}} - 1)} - 1 \right\}. \end{align*}

With this estimate of $\gamma ^{2}$, Chao and Lee [16] obtained 


\begin{align*} & \hat{S}_{\text{ACE}} = S_{\text{abund}} + \frac{S_{\text{rare}}}{\hat{C}_{\text{rare}}} + \hat{\gamma}^{2} \, \frac{f_{1}}{\hat{C}_{\text{rare}}} \;. \end{align*}

We point out again that the estimate is sensitive to the rareness abundance threshold $T$.

#### Poisson estimator (PO)

The PO estimator has already been briefly introduced above: We assume that each $(a_{i})_{1 \leq i \leq S_{\text{obs}}}$ is drawn from a Poisson distribution with an unknown parameter $\lambda>0$. We estimate $\lambda $ from the zero-truncated distribution as $\hat{\lambda }$ and then use equation (1) to estimate 


\begin{align*} & \hat{S} = S_{\text{obs}} / (1 - \text{e}^{-\hat{\lambda}}) \,. \end{align*}

We now provide details on the estimation of $\lambda $ using the maximum likelihood approach on the zero-truncated Poisson distribution, where for $k \ge 1$, 


\begin{align*} & {\mathbb{P}}[X=k] = \frac{\text{e}^{-\lambda} \lambda^{k}}{k! \, (1 - \text{e}^{-\lambda})} \,. \end{align*}

For a sample of size $n$ and observed species abundances $(a_{1}, a_{2}, \dots , a_{S_{\text{obs}}})$, the likelihood function is given by 


\begin{align*} & L(\lambda) = \prod_{i=1}^{S_{\text{obs}}} \frac{\text{e}^{-\lambda} \lambda^{a_{i}}}{a_{i}! \, (1 - \text{e}^{-\lambda})} \,. \end{align*}

It follows that the MLE must satisfy 


\begin{align*} & \frac{\hat{\lambda}}{1-e^{-\hat{\lambda}}} = \frac{n}{S_{\text{obs}}}, \end{align*}

which can be solved numerically for $\hat{\lambda }$ [31].

Instead of considering all species to estimate $\hat{\lambda }$, the sample may first be restricted to rare species with an abundance bounded by a user-defined threshold $T$ (default $T=10$), with the same implications as for the ACE estimator.

#### Chao 1 estimator (Chao 1 , Chao 1-BC)

As introduced in Section 2.3, Chao [7] proposed a *non-parametric* estimator that can be derived under the assumption that the observed species counts follow a Poisson distribution with equal detection rate $\lambda $. The estimator is given by 


\begin{align*} \hat{S}_{\text{Chao 1 }} = S_{\text{obs}} + \frac{f_{1}^{2}}{2 f_{2}} \,. \end{align*}

Chao [7] proved that the estimator yields a lower bound of the true richness for $n \to \infty $ under both multinomial and Poisson models. The lower bound can be derived based on the monotonicity of the ratio of consecutive probabilities [32, 33].

Since the Chao 1 estimator is undefined for $f_{2} = 0$, it has been replaced in later work by a bias-corrected version [29], given by 


\begin{align*} \hat{S}_{\text{Chao 1-BC}} = S_{\text{obs}} + \frac{f_{1} (f_{1} - 1)}{2 (f_{2}+1)} \;. \end{align*}

Its derivation requires additional assumptions, including species homogeneity, which often do not hold in practice [34].

A hybrid form is to use $\hat{S}_{\text{Chao 1}}$ for $f_{2}> 0$ and the bias-corrected term $S_{\text{obs}} + f_{1} (f_{1}-1) / 2$ if $f_{2}=0$.

#### Gamma-Poisson mixture estimator (GPM)

Under the assumption that the detection rate varies between species, the Poisson parameter $\lambda $ is itself a random variable. If we assume that the species-specific $\lambda _{i}$ are drawn from a Gamma distribution, then the observed species counts follow a Gamma-Poisson mixture distribution, which is a common assumption of many richness estimators [17–19].

The marginal distribution of a Gamma-Poisson mixture model with parameters $\alpha $ and $\beta $ is given for $x = 0, 1, 2, \dots $ by 


\begin{align*} P(X = x) = \frac{\Gamma(\alpha + x)}{x! \, \Gamma(\alpha)} \left(\frac{\beta}{\beta+1}\right)^\alpha \left( \frac{1}{\beta+1}\right)^{x}. \end{align*}

Under the zero-truncated Gamma-Poisson mixture model, the probability $p_{k}$ to observe a species exactly $k$ times is given by $P(X=k) / (1 - P(X=0))$.

As introduced in Section 2.3, the Horvitz–Thompson richness estimator is given by 


\begin{align*} & \hat{S}_{GP} = \frac{S_{\text{obs}}}{1-P(X=0)} = \frac{S_{\text{obs}}}{\left(\frac{\beta}{\beta+1}\right)^\alpha} \,, \end{align*}

which requires the estimation of $\alpha $ and $\beta $.

The observed frequencies $f_{k}$ follow a multinomial distribution with total sum $S_{\text{obs}}$ and probabilities $(p_{k})_{k \geq 1}$. Hence, we may solve for $\alpha $ and $\beta $ by maximizing the likelihood function 


\begin{align*} & L(\alpha, \beta) = \frac{S_{\text{obs}}!}{\prod_{k \geq 1} f_{k}!} \cdot \prod_{k \geq 1} p_{k}^{f_{k}} \,. \end{align*}

For detailed information about deriving the MLE for a Gamma-Poisson mixture distribution, we refer the reader to the paper by Chiu [19].

#### Chao and bunge estimator (CB)

Chao and Bunge [17] proposed an estimator that has a *non-parametric* form, but the optimality criteria hold under a Gamma-Poisson mixture model.

For a sample with observed richness $S_{obs}$ and number of individuals for each species in the sample given by $(a_{i})_{1 \leq i \leq S_{\text{obs}}}$, the Chao and Bunge estimator is given by 


(2) \begin{align*} & \hat{S}_{\text{CB}} = \frac{1}{\hat{\theta}} \sum_{k \geq 2} f_{k} \quad \text{ with} \quad \hat{\theta} = 1 - \frac{f_{1}}{n} \cdot \sum_{i=1}^{S_{\text{obs}}}\, a_{i}^{2} \,,\end{align*}

which is based on a consistent estimator for the expected value of $f_{0}$ under a Gamma-Poisson mixture model.

#### Lanumteang and Böhning estimator (LB)

Lanumteang and Böhning [18] derived a species richness estimator by computing a Taylor expansion over the $\log $-ratios $\log (j \, p_{j} / p_{j-1}) $, where $p_{j}$ has a Gamma-Poisson mixture distribution. Solving the equations in $j=1, 2$ for $f_{0}$ allows us to derive an estimate for $f_{0}$ that is *non-parametric* in form; given by 


\begin{align*} \hat{S}_{\text{LB}} = S_{\text{obs}} + \frac{3 f_{1}^{3} \cdot f_{3}}{4 f_{2}^{3}} \;. \end{align*}

#### Chiu estimator (Chiu)

Chiu [19] proposed a moment estimator for a Gamma-Poisson mixture model that estimates the parameters based on the expected values for the number of unseen species, singletons, doubletons, and tripletons. The derived point estimate to predict species richness is given by 


\begin{align*} \hat{S}_{\text{Chiu}} = S_{\text{obs}} + \hat{f}_{0} \cdot \left( 2 - \mathit{clip}\left( \frac{2 f^{\prime 2}_{2}}{3 f^{\prime}_{1} f^{\prime}_{3}} \right)\right) \,, \end{align*}

where $\hat{f_{0}}$ is estimated using the Chao 1 estimator, $f^{\prime}_{i}:= \max (1, f_{i})$, and $\mathit{clip}(A):= \min (\max (1/2, A), 1)$, i.e. $A$ clipped to the interval $[1/2, 1]$.

#### Objective Bayesian estimator (OB-PO, OB-NB, OB-G, OB-MG)

Under the assumption that the species abundances follow a certain parametric probability distribution, the parameters may alternatively be estimated using Bayesian statistics instead of maximum likelihood approaches, for example using Bayesian estimators that place an objective prior on the number of species and their frequency distributions.

Barger and Bunge [20] suggest to use reference priors, which maximize the expected entropy. In this review, we evaluated the Bayesian estimators for a Poisson (OB-PO), negative binomial (OB-NB), geometric (OB-G), and mixed geometric (OB-MG) distribution.

#### Recon

To estimate diversity of B- and T-cell repertoires, Kaplinsky and Arnaout [14] developed Recon, a maximum likelihood approach that makes no parametric assumption about the frequency distribution.

The estimator is calculated using an expectation-maximization approach that adds in each iteration new parameters until further parameters would lead to overfitting. The algorithm starts from a uniform frequency distribution, i.e. all species have the same number of individuals in the population. In each iteration, a new species frequency is added and the respective species counts and relative frequencies are fitted by maximum likelihood. Apart from species richness, the predicted frequency distribution may be used to estimate other diversity measures, such as entropy, the Gini–Simpson index, or Hill numbers.

#### Valiant

Valiant and Valiant [11] developed a linear program that estimates the shape of the unobserved portion of the frequency distribution. They show that their approach yields accurate results for various natural distributions if the sample size is at least in $O\left ( S / \log S\right )$ for a population with $S$ distinct species.

The algorithm is a combination of two linear programs. The first linear program searches a histogram whose expectation is closest to the observed frequency distribution. The second linear program optimizes the objective of finding a histogram that has minimal support size under the constraint that the new histogram has a similar distance to the observed frequency distribution as the one obtained by the first linear program. The coefficients to calculate the expected frequencies are computed using Poisson probabilities.

For the evaluation, we have increased the maximum number of iterations to $10000$.

#### Breakaway

Willis and Bunge [12] estimate species richness using a heteroscedastic, correlated nonlinear regression model to fit ratios of consecutive frequencies. By fitting a rational to the ratios of the form $f_{j+1} / f_{j}$ as a function of $j$, the estimate for the number of unseen elements $f_{0}$ is given by projecting the fitted function to $0$.

Since a robust estimate for the number of missing species requires an accurate number of singletons, Willis [35] enhanced the previous approach by predicting both the number of unobserved elements and the number of singletons, called Breakaway-nof1.

#### TES

Zou *et al.* [21] proposed to estimate the total species richness by fitting two asymptotic-parametric models to the probability-based rarefaction curves. For the first model, the expected value for the total species richness is computed under a hypergeometric sampling model, and for the second model under a multinomial sampling model. The two parametric models were previously introduced by Hurlbert [36] and Smith and Grassle [37], respectively. To estimate the total species richness, a four-parameter Weibull-logistic regression model is fitted to the change in expected value for increasing sample sizes. The species richness estimate is given by the asymptote of the fitted function. If the sample is too small to successfully fit a Weibull-logistic model, a three-parameter logistic regression model is fitted instead. The final richness estimate is given by the mean value of the asymptote under a hypergeometric and multinomial model.

#### Upsampling estimators

The previous estimators attempt to estimate the species richness $S$ of the full population without knowing its size $N$, even allowing infinite $N$, but assume that $S$ is finite. In contrast, the following estimators are given the population size $N$ as additional input, and therefore do an *upsampling* or *extrapolation* task (from observed richness $S_{\text{obs}}$ and $(f_{k})_{k\ge 1}$ with $n$ individuals to the unknown $S$ with $N$ individuals). The difficulty of the upsampling problem increases with the ratio $N/n$.

#### iNEXT

The iNEXT R package [22] provides a combined framework to interpolate (i.e. compute the rarefaction curve) and extrapolate Hill numbers of several diversity orders. For abundance data, the Hill numbers of order $q$ are defined for a sample with relative abundances $p=(p_{1}, \dots , p_{S_{\text{obs}}})$ as 


\begin{align*} ^{q}D = \left(\sum_{i=1}^{S_{\text{obs}}} p_{i}^{q}\right)^{1/(1-q)}. \end{align*}

Hence, the sample species richness corresponds to Hill numbers of diversity order $0$, completely disregarding relative species abundances. To extrapolate Hill numbers from an initial sample of size $n$ to a larger sample of size $n + m$, Chao *et al.* [38] introduced extrapolated diversity estimators $^{q} \hat{D}(n + m)$ for any $m> 0$. For diversity order $0$, the size-based extrapolated species richness for an enlarged sample of size $n+m$ is given by 


\begin{align*} ^{q} \hat{D}(n + m) = S_{\text{obs}} + \hat{f}_{0} \left(1 - \left(1 - \frac{f_{1}}{n \hat{f}_{0} + f_{1}}\right)^{m}\right) \;, \end{align*}

where $\hat{f}_{0}$ can be any proper estimator for $f_{0}$, e.g. the Chao 1 estimator for $f_{0}$. However, as already noted by Colwell *et al.* [13], the above estimator is only reliable for $m < n$ (see Chao *et al.* [38] for more detailed information).

#### Good–Toulmin estimator (EF-GT, PO-GT)

To estimate how many unseen species may be expected in a next sample, Good and Toulmin [30] proposed an estimate that is based on the assumption that the capture rates follow a Poisson distribution. Since the probability of observing a new species is given by the probability that a species was not seen in the first but was seen in the second sample, the expected number $E$ of new species one would see in a second sample *of same size* is given for a population with $S$ species by 


(3) \begin{align*} E &= \sum_{s=1}^{S} P_{\lambda_{s}}(X = 0) \cdot (1-P_{\lambda_{s}}(X = 0)) \end{align*}

(4) \begin{align*} &= \sum_{s=1}^{S} e^{-\lambda_{s}} \cdot (1-e^{-\lambda_{s}}) \,, \end{align*}

where $P_{\lambda _{s}}(X = i)$ denotes the probability to observe exactly $i$ individuals from species $s$ under a Poisson distribution with parameter $\lambda _{s}$. The series expansion of the second term is given by 


\begin{align*} & (1-e^{-\lambda_{s}}) = \sum_{i=1}^\infty \frac{(-1)^{i-1} \lambda_{s}}{i!} \,, \end{align*}

which may be used to rewrite equation (4) as 


\begin{align*} E &= \sum_{s=1}^{S} e^{-\lambda_{s}} \cdot \sum_{i=1}^\infty \frac{(-1)^{i-1} \lambda_{s}}{i!}\\ &= \sum_{i=1}^\infty\, (-1)^{i-1} \cdot \sum_{s=1}^{S} P_{\lambda_{s}}(X = i) \,. \end{align*}

Good and Toulmin [30] approximate the sum $\sum _{s=1}^{S} P_{\lambda _{s}}(X = i)$ by the observed $f_{i}$ and generalize the procedure to general sizes $m$ of the second sample. They obtain an estimate for the number of unseen elements $\hat{U}$ in a new random sample of size $m$ from a sample of size $n$: 


(5) \begin{align*} \hat{U}_{\text{GT}} = - \sum_{i\geq 1} (-m/n)^{i}\, f_{i}\end{align*}

with ratio $t = m/n$. Note that $t=1$, as derived above, corresponds to upsampling by a factor of 2, where the second sample has the same size as the first, and $t=99$ corresponds to 100x upsampling. They showed that the above formula is a nearly unbiased *non-parametric* estimator of $U$ for all $t \leq 1$, but the convergence of the series is not guaranteed for $t> 1$.

One possibility to achieve convergence for $t> 1$ is to perform so-called probabilistic smoothing, yielding new estimators of the form 


\begin{align*} \hat{U}_{L} = - \sum_{i \geq 1}\, (-t)^{i}\, P(L \geq i)\, f_{i} \;, \end{align*}

where the discrete random variable $L$ can follow an arbitrary discrete distribution.

Efron and Thisted [39] proposed Binomial smoothing (GT-ET), such that 


\begin{align*} P(L \geq i) = \sum_{j=i}^{k} \binom{k}{j}\, p^{j} \, (1-p)^{k-j} \;, \end{align*}

with $p = \frac{1}{1+t}$, and $k$ being a tuning parameter. We use $k:= \lfloor \frac{1}{2} \log _{2} \big ( \frac{nt^{2}}{t-1} \big )\rfloor $, which has been shown by Orlitsky *et al.* [23] to lead to the best convergence rate.

As an alternative, Poisson smoothing (GT-PO) uses 


\begin{align*} P(L \geq i) = 1 - \sum_{j=0}^{i-1} \frac{\lambda^{j} \, e^{-\lambda}}{j!} \end{align*}

with $\lambda = \frac{1}{2t} \cdot \big ( \frac{n \, (t+1)^{2}}{t-1} \big )$ [23].

The resulting estimates $\hat{U}$ are in fact estimates of $f_{0}$; thus the richness estimator is $\hat{S} = S_{\text{obs}} + \hat{U}$. This also holds for the following estimators, which use more complex procedures to estimate the number of unseen species.

#### PreSeq

To approximate the molecular complexity of sequencing libraries, Daley and Smith [24] introduced the idea of using rational function approximation to increase the radius of convergence of the Good–Toulmin power series, given by equation (4). Rational function approximation increases the radius of convergence for divergent series, in particular for alternating power series such as the Good–Toulmin power series. This approach allows to predict the species richness for samples that are several orders of magnitudes larger than the reference sample.

In addition, PreSeq first applies the Euler transform to equation (5), as proposed by Good [2], yielding the power series 


\begin{align*} U_{\text{ET}} = \sum_{i \geq 1}\, (-1)^{i-1} \, (t-1)^{i} \, f_{i} \,. \end{align*}

By transforming the variable $t$ of the power series, PreSeq considers a larger class for the rational function approximation, but under the constraint that the first coefficients are equal to the coefficients of the original power series, hence trusting the original series more in the neighborhood around $t=1$ [24].

#### Pitman/Ewens sampling formula (PSF)

The Pitman sampling distribution, a two-parametric generalization of the Ewens sampling formula, is a common sampling model that assumes an infinite sampling universe [25]. The urn representation of the Pitmam sampling formula is given by the Hoppe urn model. The formula calculates the probability of observing an integer partition of $n$, where a partition is assumed to be random and exchangeable. The set of valid integer partitions is given by $P_{n} = \{ (f_{k})_{k=1}^{n} \;|\; f_{k} \in \mathbb{N}_{0}, \sum _{k=1}^{n}\, k\, f_{k} = n \}$. The probability of a partition $f$ under a Pitman sampling model with parameters $ \alpha \in ]0, 1] $ and $\theta \in [-\alpha , \infty ]$ is defined as 


\begin{align*} P_{\alpha, \theta}(f) &= n! \cdot \frac{(\theta)_{S_{\text{obs}};\alpha}}{(\theta)_{n}} \cdot \prod_{k=1}^{n}\, \Big[\frac{(1-\alpha)_{k-1}}{k!} \Big]^{f_{k}} \cdot \frac{1}{f_{k}!} \,, \\ \text{where } (\theta)_{i;\alpha} &:= \begin{cases} 1 & (i = 0) \,,\\ \prod_{j=0}^{i-1}\, (\theta + j \alpha) & (i=1, 2, \dots) \,, \end{cases} \\ (\theta)_{i} &:= (\theta)_{i;1} = \theta (\theta + 1) \cdots (\theta + i - 1) \, \end{align*}

[25]. To estimate species richness, the parameters $\alpha $ and $\theta $ of the Pitman sampling distribution can be estimated using maximum likelihood estimation. Given $\alpha $ and $\theta $, the expected number of additional elements $\hat{U}_{\text{PS}}$ in a next sample of size $m$ is given by [40] 


\begin{align*} & \hat{U}_{\text{PS}} = \left( S_{\text{obs}} + \frac{\theta}{\alpha} \right) \left( \frac{(\theta + n + \alpha)_{m}}{(\theta+n)_{m}} - 1 \right)\,. \end{align*}

#### DivE

Rarefaction curves show the number of distinct elements as a function of the sample size and are a common method in diversity estimation to analyse species richness via nested subsampling. Traditional curve fitting approaches fit a parametric asymptotic function, such as a negative exponential or logistic function, to the rarefaction curve [41]. The species richness is then given by the asymptote of the function.

Laydon *et al.* [26] extended this idea by fitting a list of 58 mathematical function classes to the rarefaction curves and accumulating the results of the five best fitting classes. Instead of computing the asymptote, the predicted species richness is given by extrapolating each function to the desired sample size. Since testing all mathematical functions is very compute intensive, we restrict our evaluation to previously suggested functions: the logistic, negative exponential, logarithmic, hyperbolic, and Hill function families.

#### RichnEst

Schröder and Rahmann [10] developed a linear program (LP) to estimate the molecular complexity or duplication rate of sequencing experiments from a small sample. The linear program searches for plausible frequency vectors of the whole population by minimizing the distance between the expected frequency vector and the observed frequency vector of the sample.

Assuming that the sample is drawn randomly, the probability that a species with $K$ individuals in the complete population of size $N$ is observed exactly $k$ times in a sample of size $n$ follows a hypergeometric distribution, which allows us to compute the expected frequency vector, assuming a population frequency vector [10]. The LP formulation is used to invert this forward downsampling process.

## Evaluation

### Data simulation

**Figure 2:**
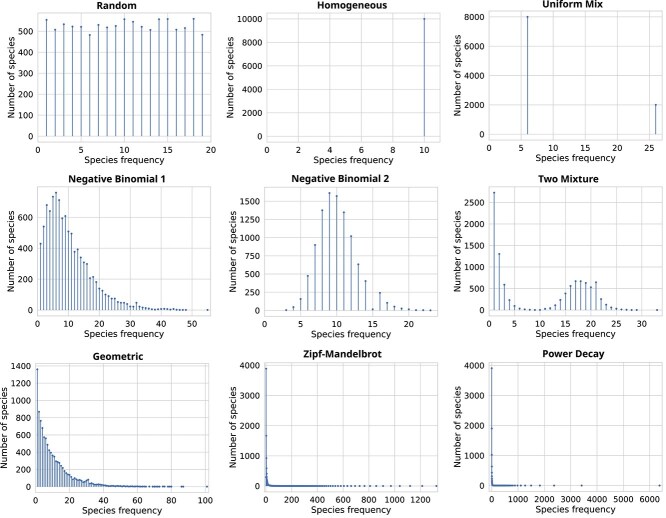
Simulated data. Each plot shows the species frequency distribution of a population with $10^{5}$ individuals and $10^{4}$ distinct species under different population models, described in Section 3.1.

The richness estimators are evaluated on simulated data with nine different species compositions (Fig. 2). We consider samples containing $1\%, 3\%, 5\%$, as well as $10\%, 20\%,..., 90\%$ of the population. For each test case, a sample is drawn $20$ times, resulting in a total of $2160$ test cases.

For each species composition, the relative abundances for a population with $N = 10^{5}$ individuals and $S=10^{4}$ distinct species are given by $(p_{1}, p_{2}, \dots , p_{S}) = (c A_{1}, c A_{2}, \dots , c A_{S})$, such that $\sum _{i=1}^{S}\, p_{i} = 1$ and $\sum _{i=1}^{S}\, A_{i} = N$, where the $A_{i}$ are the absolute population abundances and $c = 1/N$ [19]. Below, we specify $p_{i}$ or $A_{i}$ of different population composition models. Data simulation and evaluation was automated using Snakemake [42].

#### Random model

For $i = 1, \dots , S$, we draw $p_{i}$ from the uniform distribution on $[0.02,0.98]$, normalized such that $\sum _{i=1}^{S}\, p_{i}=1$. We then multiply the $p_{i}$ by the population size $N$ to obtain the abundances $a_{i}$, with an average of $10$.

#### Homogeneous model

For $i = 1, \dots , S$, we set $p_{i} = 1/S$. Thus, for $N = 10^{5}$ and $S=10^{4}$, each species has exactly 10 individuals.

#### Uniform mixture model

For one fifth of the species, $i = 1, \dots , S/5$, we set $p_{i} = 5/(2S)$, and for the remaining 4/5 of the species, $i = S/5 + 1, \dots , S$, we set $p_{i} = 5/(8S)$ (assuming that $S$ is a multiple of 5).

#### Negative binomial models

We use two different models. For $i = 1, \dots , S$, we set $p_{i} = c A^{\prime}_{i}$, where $A^{\prime}_{i}$ is a random sample drawn from a negative binomial distribution either with parameters $r=2$ and $p=0.02$ (model 1), or with $r=20$ and $p=0.2$ (model 2).

#### Two mixture model

We set $p_{i} = c A^{\prime}_{i}$, where $A^{\prime}_{i}$ is a random sample drawn from a negative binomial distribution with parameters $r=2$ and $p=0.1$ for one half of the species and with parameters $r=50$ and $p=0.2$ for the other half of the species.

#### Geometric model

For $i = 1, \dots , S$, we set $p_{i} = c A^{\prime}_{i}$, where $A^{\prime}_{i}$ is a random sample drawn from a geometric distribution with parameter $p = 1/S$.

#### Power decay model

For $i = 1, \dots , S$, we set $p_{i} = c / i^{0.9}$, where $c$ is the proper normalization constant.

#### Zipf–Mandelbrot model

For $i = 1, \dots , S$, we set $p_{i} = c / (i+10)$, where $c$ is the proper normalization constant.

### Real datasets

We apply the tools to publicly available immune repertoire, microbiome, and reef fish datasets.

The repertoire sequencing data published by Shugay *et al.* [[43], VDJTools Examples] contains targeted sequencing of V(D)J genes, which code for distinct antibodies and T-cell receptors. The unique arrangement of V(D)J segments is called the clonotype of a cell. The estimation of clonotype richness enables a high level analysis of immune repertoire diversity. We apply the tools to 58 available samples, for which we create 10 subsamples for each of 6 subsampling rates of 1%, 3%, 5%, 10%, 20%, and 30%, resulting in 3480 test cases.

To estimate microbiome diversity, we apply the tools to 20 metagenomic datasets from Durazzi *et al.* [[44], MG-Rast database], which contain the abundance of bacterial strains in the chicken gut at different taxonomic levels. In our evaluation, we estimate bacterial richness based on the taxonomic classification at the genus level. For each dataset, we create the same $10\times 6$ subsample types as for the immune repertoires, resulting in $1200$ test cases.

In addition, we evaluate the methods on an ecological dataset of global reef fish communities [45, Fish dataset] from [45]. For each fish species, we sum up the species abundances of different size classes. The methods are evaluated on 10 repeated subsamples containing 1%, 3%, 5%, 10%, 20%, and 30% of the complete fish dataset.

### Results

The evaluated methods require as input either the observed abundance vector $(a_{i})_{1 \leq i \leq S_{\text{obs}}}$, i.e. the number of times each species was observed in the sample, or the frequency vector $(f_{k})_{k \geq 1}$, i.e. the number of species occurring exactly $k$ times in the sample, and output a point estimate and sometimes a confidence interval of the population species richness. Since not all methods compute a confidence interval, we measure the accuracy based on the point estimates.

On simulated data, we evaluate all methods with respect to (1) proportion of crashes, (2) proportion of outliers, (3) point estimation accuracy, and (4) computational resource requirements. Next, we evaluate the point estimation accuracy for V(D)J, microbiome, and fish subsamples.

#### Proportion of unsolved problems

**Figure 3:**
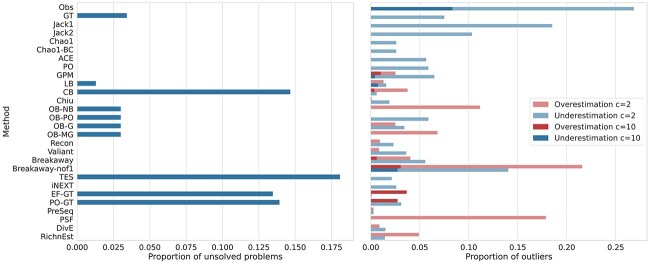
**A** For each tool, the proportion of unsolved problems is shown. Lower is better; zero is desirable. **B** Proportion of outliers for each tool. For constant $c\in \{2,10\}$, each point estimate that is smaller than $S/c$ or larger than $cS$ is assumed to be an outlier that heavily under- or overestimates the true population richness, respectively. Lower is better.

Figure 3A shows the proportion of unsolved test problems for each method. A test problem is unsolved if the tool either crashed or failed to converge to a solution. Most of the tools are able to compute a point estimate for all problems, except for the objective Bayesian estimators, TES, and smoothed Good–Toulmin estimators, which sometimes fail to converge. In addition, the Good–Turing and Lanumteang–Böhning estimator fail if $f_{1}$ or $f_{2}$ are zero, respectively. A similar problem holds for the Chao–Bunge estimator if $\hat{\theta }=0$ in equation (2). For detailed information about the proportion of unsolved problem per subsampling rate and population, see Supplementary Figure S.2.

#### Proportion of outliers

We compare the number of outliers per tool, where we consider an estimate to be an outlier if $\hat{S} < S/c$ or $\hat{S}> cS$, for a constant $c>0$. Figure 3B shows the proportion of outliers for $c=10$ and $c=2$.

As expected, the observed species richness (Obs) often strongly underestimates the true richness. For several test cases, Breakaway(-nof1) and the maximum-likelihood based Poisson and Gamma-Poisson estimators strongly over- and underestimate the true richness. In addition, the Pitman sampling formula and the smoothed Good–Toulmin estimators strongly overestimate the true richness for small sampling rates. For the Good–Toulmin estimators, this may be caused by divergence of the power series. In general, simple estimators, such as Chao1, usually underestimate the true richness, while more complex estimators both under- and overestimate the true richness (see Figure 3B). Moreover, all tools have most outliers for small sampling rates (1% to 5%). For larger sampling rates, populations with a Power Decay and Zipf–Mandelbrot distribution are most challenging (see Supplementary Figure S.3).

#### Estimation accuracy

**Figure 4:**
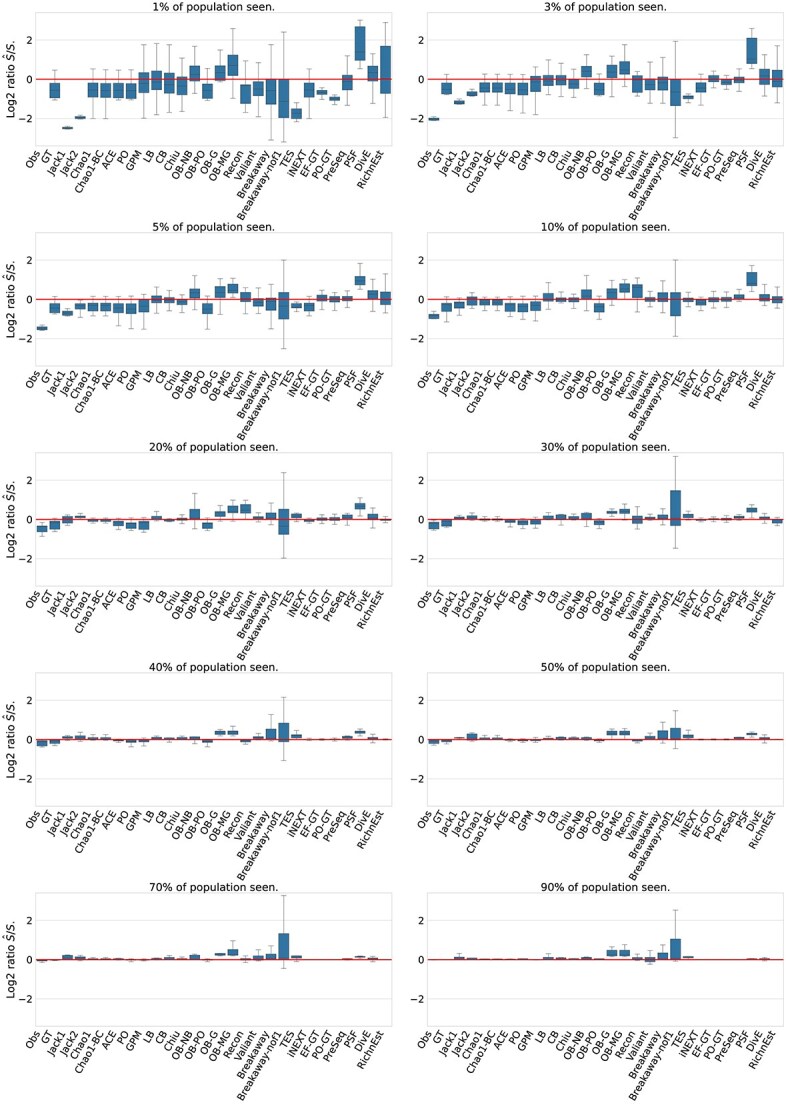
Box plots of estimation accuracy on simulated data. Shown are $\log _{2}$ ratios between the estimated species richness and the true species richness for different subsampling rates (panels), combined over all population models. If the prediction is correct, $\log _{2} \hat{S}/S = 0$ (red horizontal line). If $\log _{2} \hat{S}/S> 0$, the estimator overestimates the true richness and if $\log _{2} \hat{S} / S < 0$, the estimator underestimates the true richness. Failures and outliers (using $c=10$; see Fig. 3) are not included in this data.

Figure 4 gives a more detailed overview of the estimation accuracy. Each panel corresponds to a sampling rate. Each box plot shows the deviation of the point estimate from the true richness across all population models for one method. Individual plots for each population model are provided in the supplementary figures.

**Figure 5:**
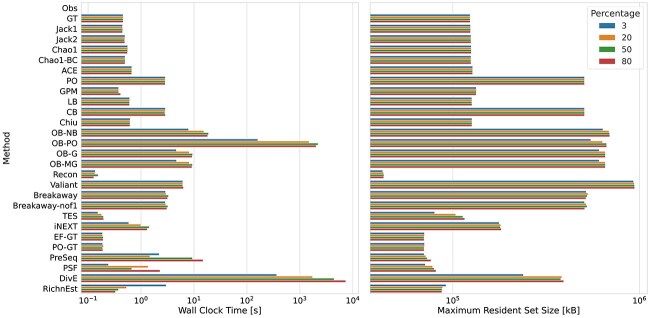
Computational resource requirements: **A** Wall clock time in seconds; **B** Memory usage in kilobytes. The benchmarks for a sample containing 3%, 20%, 50%, and 80% of the total population were averaged over three runs for populations with a negative binomial ($r=20$ and $p=0.2$) and Zipf–Mandelbrot frequency distribution on a AMD Ryzen 9 5950X 16-Core processor with a maximum CPU clock speed of 5.1 GHz.

In general, the estimation problem is more challenging when only a small portion of the population has been observed, resulting in an extreme underestimation of the true richness by many tools, such as the Chao 1 estimator, the ACE estimator, TES, or Valiant. When the sample contains more than $50\%$ of the population, asymptotic estimators overestimate the true richness for some species compositions. For example, the Chao 1, ACE, and Chiu estimators, which are often referred to as a lower bounds, yield accurate lower bounds close to the true species richness for most species compositions, but overestimate the species richness for the two mixture model if the sample contains more than $40\%$ of the population (Supplementary Figure S.8).

The non-parametric and Gamma-Poisson mixture estimators give accurate results for populations with a negative binomial and homogeneous frequency distribution, but tend to underestimate the true richness for populations under a geometric, Zipf–Mandelbrot or power decay model (Supplementary Figures S.4–S.12), with the Chao 1 , ACE, and Chiu estimators being among the most accurate methods.

Breakaway and Breakaway-nof1 both over- and underestimate the true richness and have large estimation intervals for repeated samples (Supplementary Figure S.7). Recon and the Bayesian Geometric and Mixed Geometric estimators tend to overestimate the true richness. Even for a population with a geometric frequency distribution, the objective Bayesian geometric and mixed geometric estimators consistently overestimate the true richness (Supplementary Figure S.9).

Upsampling methods, show increasing accuracy with increasing sample size. An increased sample size means that we have smaller upsampling (extrapolation) factors or that we observed a larger fraction of the complete population.

The smoothed Good–Toulmin estimators provide accurate results for many of the evaluated problems. However, they can suffer from convergence problems, e.g. many of the problems could not be solved by the smoothed Good–Toulmin estimators if less than $30 \%$ of a population with a power decay frequency distribution was observed (see Supplementary Figure S.2). PreSeq’s approach of using rational function approximation successfully increases the convergence ratio of the power series. PreSeq is able to solve all problems and is among the best performing tools for populations with a power decay or Zipf–Mandelbrot distribution, in particular for low sampling rates (Supplementary Figure S.12). The performance of DivE is similar to PreSeq showing an increasing accuracy for larger samples. For low sampling rates ($<10\%$), the estimates of RichnEst show high variation and both over- and underestimate the true richness. For sampling rates $\geq 10\%$, RichnEst gives accurate richness estimates. Because iNEXT was developed to extrapolate to a new sample 2 to 3 times the reference sample size, it is less accurate and underestimates the species richness for lower sampling rates, but provides accurate results for sampling factors $>30\%$.

Although the Pitman sampling formula also requires an upsampling factor, it is less accurate than most population and upsampling estimators, i.e. the true richness is often overestimated (see Fig. 4).

#### Computational requirements

Running times range from under one second for tools such as the Good–Turing, Jackknife, Chao 1, and Gamma-Poisson mixture model, to an hour for the Objective Bayes Poisson estimator and several hours for DivE. Although we evaluated DivE with only 5 different mathematical function families, the running time was already significantly higher compared to the other methods. In addition, DivE’s time requirements strongly increase with increasing sample size, which makes it impractical for large datasets.

The maximum memory requirements are under 1 GB for all tools and evaluated sample sizes and largely independent of sample size.

#### Evaluation on Real Data

Methods that could not solve all problems (Good–Turing, Chao–Bunge, Objective Bayesian, smoothed Good–Toulmin, TES), had extreme outliers (Breakaway, Breakaway-nof1, Pitman sampling formula), performed less accurate for most problems (Jackknife 1 and 2, Poisson model) or have impractical computation times on large data (DivE, e.g. 120h for one V(D)J dataset and a subsampling rate of $0.3$) are not considered in the subsequent evaluation.

**Figure 6:**
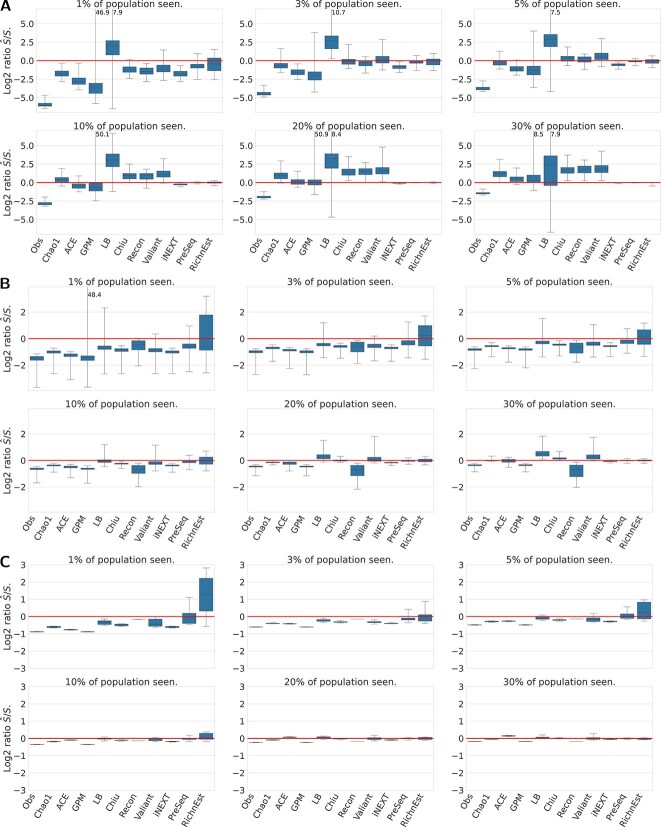
Estimation of species richness for **A** V(D)J immune repertoire data (sequence level) **B** microbiome data (Lactobacillus strains in broiler after taxonomic classification at the genus level) **C** global reef fish communities. The box plots display the distribution of $\log _{2}$ ratios between the predicted species richness and the species richness observed in the complete dataset. Numbers at the cut whiskers show the maximum deviation of the method.

Figure 6 shows the deviation of the predicted species richness from the observed species richness in the complete V(D)J sequencing, microbiome and reef fish datasets for different subsampling rates. The box plot labeled “Obs” shows the number of observed species in the subsample; it always underestimates the true richness. For the population estimators, the observed richness of the complete dataset imposes a lower bound on the true species richness, but the true population richness may be higher considering that real data never samples the whole population. The rarefaction curves for the V(D)J data indicate that the total VDJ diversity is substantially larger than the full sample richness. In contrast, the rarefaction curves for the microbiome and reef fish data suggest that the population richness is close to the sample richness (see Supplementary Figure S.13). The estimates of the upsampling estimators iNEXT, PreSeq, and RichnEst should be close to the observed richness of the complete dataset.

#### Estimating V(D)J richness

On the V(D)J data (see Fig. 6A), the lower bounds of Chao 1, ACE, and the richness estimator of the Gamma-Poisson mixture model strongly underestimate the true richness for low subsampling rates, which is consistent with the previous results that these estimators tend to strongly underestimate the true richness for compositions with many rare elements. The predictions by Chiu, Recon, and Valiant are close to the true richness of the full sample if $3\%$ of the population has been observed, but they predict a higher estimate for higher subsampling rates. Since the true richness of the immune repertoire is unknown (our 100% is in fact also only a sample of unknown proportion), the accuracy cannot be validated, but the almost linear rarefaction curves indicate that the total VDJ diversity is substantially larger than the full sample richness (see Supplementary Figure S.13). This equally holds for the Lanumteang–Böhning estimator, which is the only method that predicts an extremely higher richness for all subsampling rates. In general, for too small subsampling factors, population (asymptotic) estimators are not able to provide accurate estimates that are independent of the sample size.

Among the upsampling estimators, the point estimates of RichnEst vary more compared to iNEXT and PreSeq. For a subsampling rate of $3\%$ and $5\%$ the predictions of RichnEst are on average closer to the true richness. In particular, iNEXT underestimates the true richness due to the limited reliability when the sample size is more than doubled. All upsampling estimators show an increasing accuracy for larger subsamples.

#### Estimating microbiome richness

For the microbiome data, the true population richness can be assumed to be close to the sample richness (see rarefaction curves in Supplementary Figure S.13). Figure 6B shows that all methods underestimate the species richness for subsampling rates below $10\%$, except for RichnEst, which shows a high variation for small subsampling rates. For a subsampling rate of $10\%$ and $20\%$ only the Lanumteang–Böhning estimator and Valiant predict a higher population richness. Recon strongly underestimates the species richness. All other methods have a similar performance, with PreSeq yielding the most accurate results.

#### Estimating global reef fish richness

The results for the reef fish data are very similar to the microbiome data (see Fig. 6C): For low subsampling rates, the species richness is underestimated and with increasing subsampling rates the estimation of all methods converges to the total sample richness.

## Discussion and conclusions

The increasing applications of richness estimation, for instance estimating the diversity of immune repertoires, make the accurate estimation of species richness from a small sample an important research topic. Although a variety of richness estimators already exist, new approaches that are either specific or generally applicable to many different scenarios, are still being developed. In particular, recent methods try to tackle the challenges of undersampled and heterogeneous species compositions. We presented a methodologically focused overview of existing richness estimators and evaluated them on a wide range of populations with different species compositions.

Richness estimators are classified into population and upsampling estimators, depending on whether they require the size $N$ of a future sample as an additional parameter. In ecology, they are referred to as asymptotic and extrapolating richness estimators, respectively.

Asymptotic (population) estimators often provide lower bounds and may have a limited reliability as point estimates for strongly undersampled populations. Among the population estimators, the estimators that are non-parametric in form, like the Chao 1 , ACE, and Chiu estimator, provided the most accurate point estimates for a variety of simulated species composition, but strongly underestimated the true species richness if the population was either very heterogeneous are only a small portion of the sample had been observed. The estimates of more complex methods, like Breakaway or the Objective Bayesian estimators, often had a high variation for repeated subsamples from the same population and both strongly over- and underestimated the true richness.

Upsampling (extrapolating) estimators generally give accurate estimates of the total species richness, except for the Pitman sampling formula, which gave inaccurate results for many problems. RichnEst and iNEXT provided accurate results for large subsampling rates, but suffered from inaccuracies when the sample was too small ($<10\%$ for RichnEst and $<30\%$ for iNEXT). For small subsamples, PreSeq often outperformed the other approaches.

We observed that the accuracy on downsampled real data was comparable to the accuracy on simulated data. However, downsampling a complete population results in a clean dataset, and its properties may be very different from actual real data, such as for amplicon-based microbiome sequencing data. Data cleaning and preprocessing are common steps in microbiome analysis, but may introduce conditions that invalidate the direct use of some estimators. For example, it is common to remove singleton species from the sample, because they may be explained by misclassifications caused by sequencing errors; although some may be correct. In this case, non-parametric estimators, such as the Chao 1 and Chiu estimator, that are based on the number of singletons are not directly applicable. To solve this problem, Chiu and Chao [46] propose to use an estimate for the number of singletons instead of the sample singletons. However, recent work on gut microbiome analysis suggests to filter out all low abundance taxa to remove contamination, in particular for 16S amplicon-based sequencing data [47]. In this case, the results of most richness estimators should be treated with caution, because many estimators, such as Chao 1, Chiu, ACE, Jacknife, or iNEXT, assume that most of the information about missing species is contained in the number of low abundance species. Our study does not evaluate the performance of the considered estimators under unclean data or processed data with such biases. This remains an important topic for future work.

**Key Points**

- comprehensive review of published methods for species richness estimation on simulated artificial data and downsampled real data (immune repertoire, microbiome, and global reef fish data)
- mathematical foundations and statistical assumptions of richness estimation methods
- Heterogeneous species compositions are more challenging than homogeneous species compositions.
- Population (asymptotic) estimators, such as Chao 1 or Chiu, yield accurate lower bounds if the number of singletons in the sample is correct.
- Upsampling (extrapolating) estimators, such as PreSeq or RichnEst, allow accurate richness estimation for samples up to $10\times $ larger than the reference sample.

## Supplementary Material

supplement_bbaf158

## Data Availability

All evaluated data is available online. The V(D)J data was published in [43], the microbiome data in [44], and the reef fish data in [45]. Detailed accession numbers and download scripts are provided in the code repository (see Code availability).

## Code availability

The code to run all methods is available at https://gitlab.com/rahmannlab/speciesrichness. The repository contains code for simulating the data, download scripts for the real datasets, and Snakemake workflows to run all evaluations.
