# Supplementary material for: A comprehensive review and evaluation of species richness estimation
Source: Brief Bioinform. 2025 Apr 11;26(2):bbaf158. doi: 10.1093/bib/bbaf158 (PMC11986355; doi:10.1093/bib/bbaf158)
Supplement: supplement_bbaf158 [file supplement_bbaf158.pdf]

Supplementary Figures

A Comprehensive Review and Evaluation of Species Richness Estimation

Johanna Elena Schmitz and Sven Rahmann

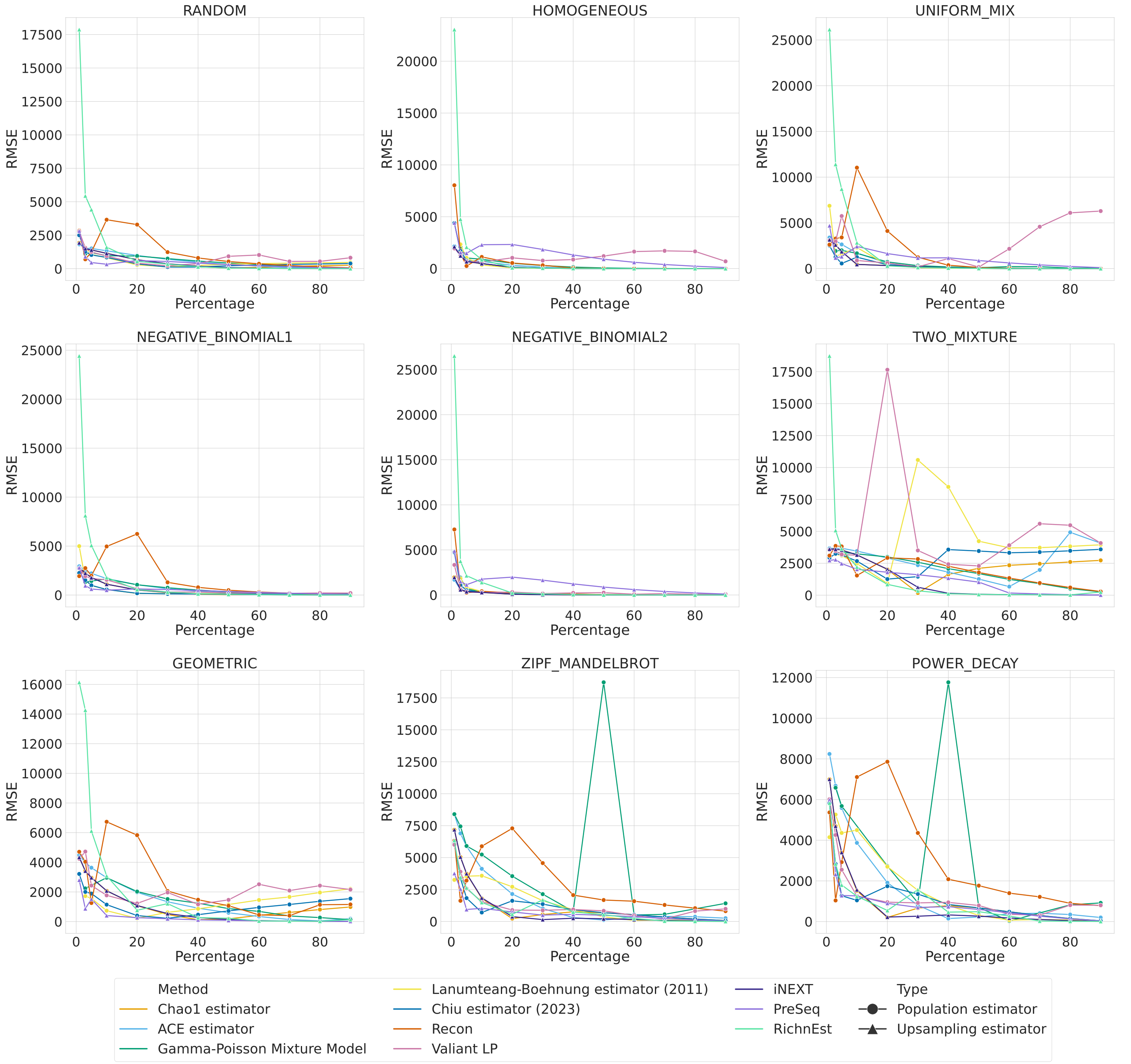

Figure 1: Root mean square error (RMSE) changes with increasing sample size across population models. The RMSE is defined for k samples with true species richness $S_1, \ldots, S_k$ and estimated species richness $\hat{S}_1, \ldots, \hat{S}_k$ as $\left(\frac{1}{k}\sum_{i=1}^{k}(\hat{S}_i - S_i)^2\right)^{1/2}$. Estimators or tools were not considered if they could not solve most problems (Good-Turing, Chao-Bunge, Objective Bayesian, smoothed Good-Toulmin), had many outliers (observed richness, Gamma-Poisson mixture model, Breakaway-nof1, UnseenEst) or performed badly for most problems (Jackknife 1 and 2, Pitman sampling formula).

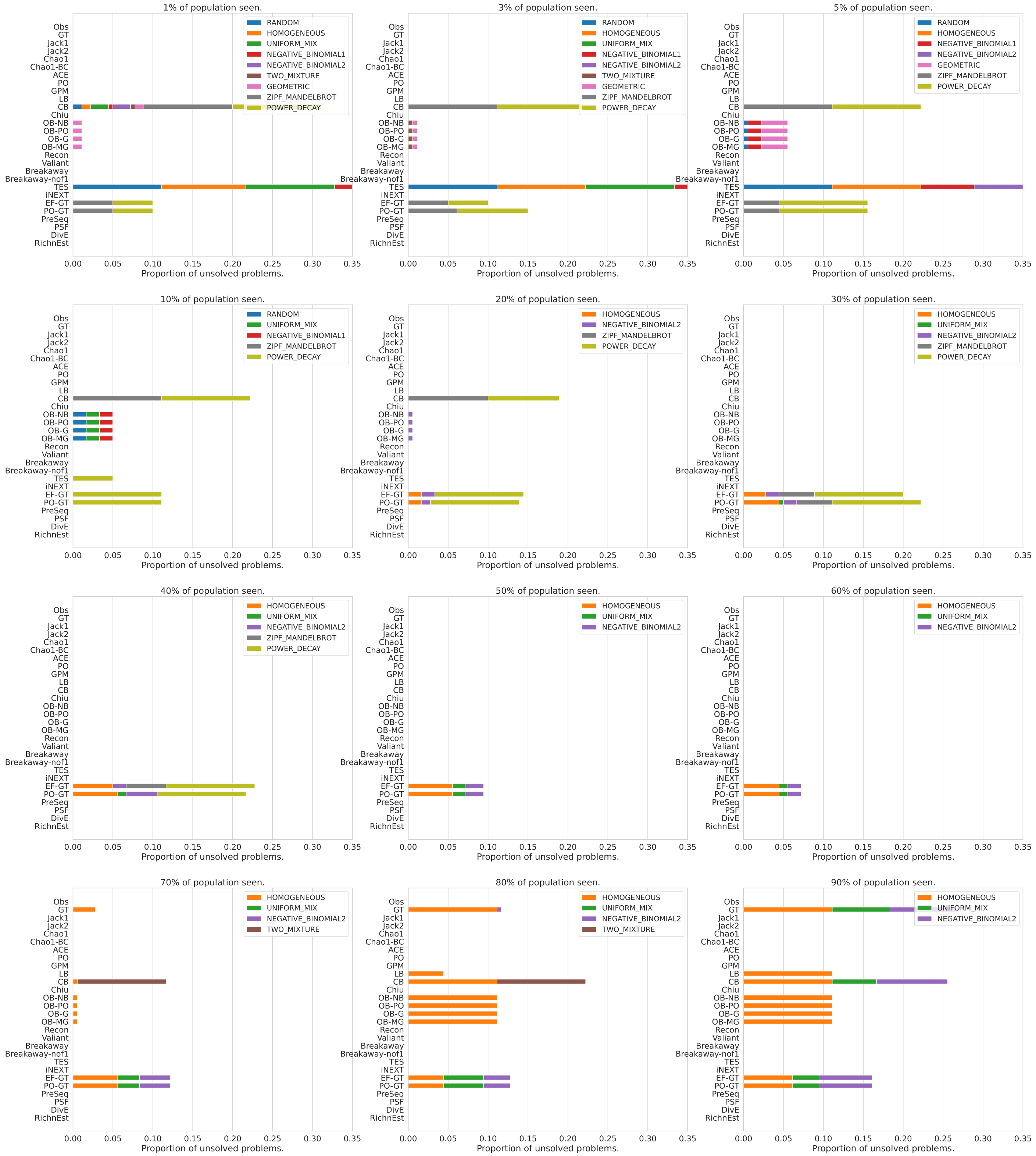

Figure 2: Proportion of unsolved problems for each method. Each subplot shows the proportion of unsolved problems for one subsampling rate. The colored bars further divide the unsolved problem by population model. The unsolvable problems vary for the tools and depend on both the subsampling rate and the population model

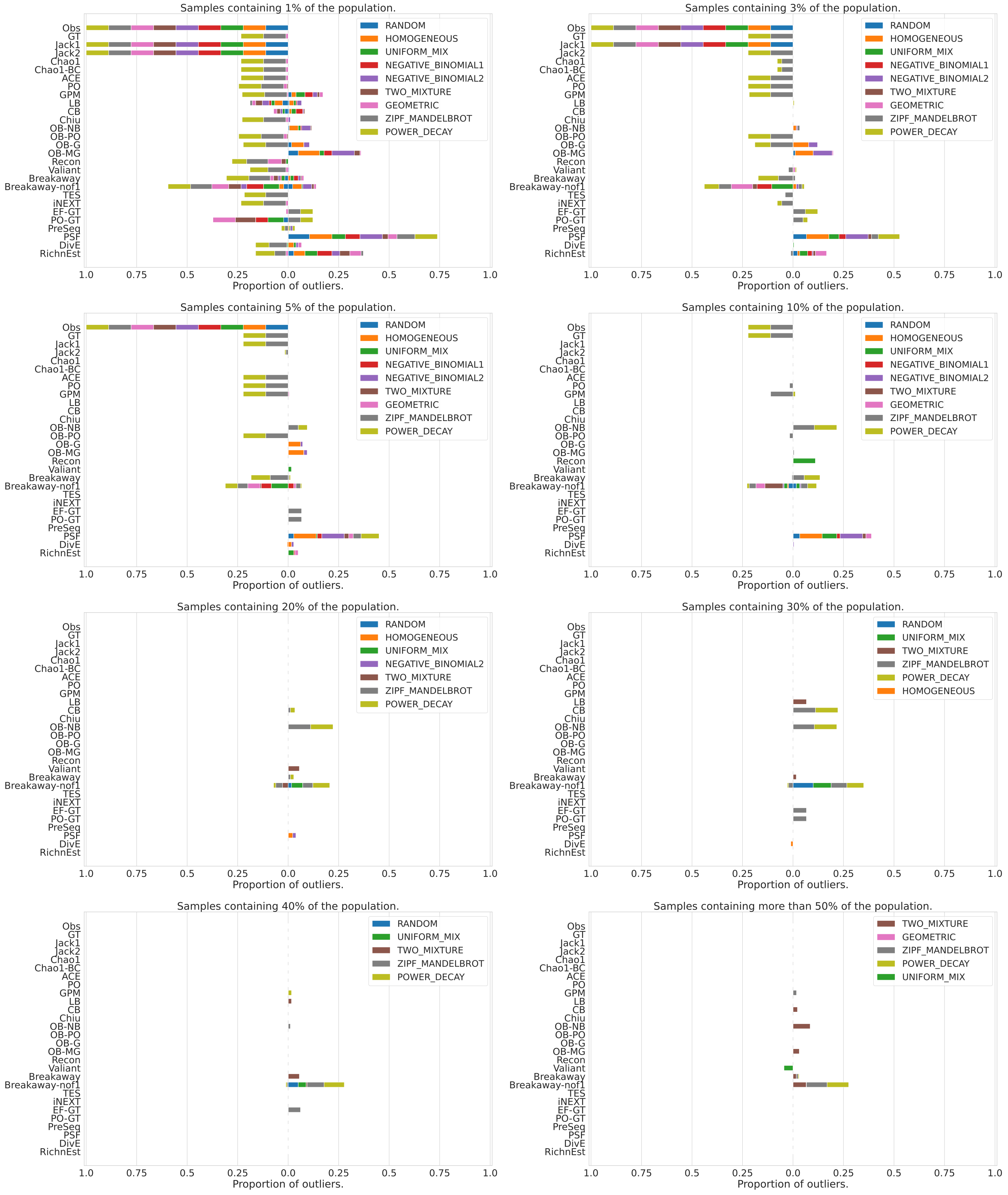

Figure 3: Proportion of outliers for each tool and each subsampling rate. The colored bars further divide the outliers by population model. To the left are the outliers that underestimate and to the right are the outliers that overestimate the true richness. An estimate is assumed to be an outlier if it is smaller than S/c or larger than $c \cdot S$. For this plot, we used $c = 2$. The 'hard' problems with most outliers occur for subsampling rates below 0.1.

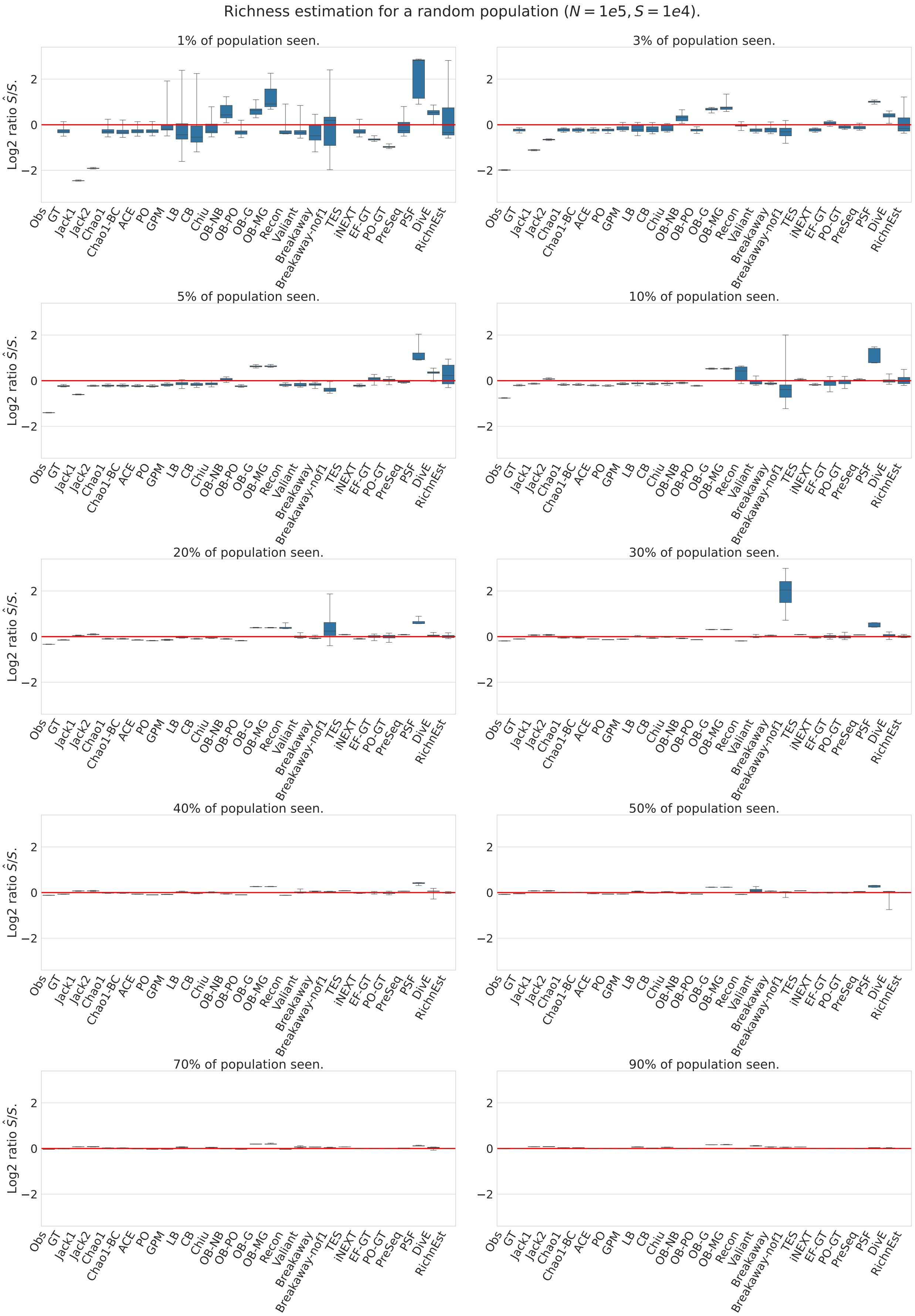

Figure 4: Estimation accuracy for the *random* population model. Boxplots display $\log_2$ ratios between the estimated species richness $\hat{S}$ and the true species richness S. If the prediction is correct, $\log_2(\hat{S}/S) = 0$. If $\log_2(\hat{S}/S) > 0$, the estimator overestimates the true richness and if $\log_2(\hat{S}/S) < 0$, the estimator underestimates the true richness. Outliers have been removed (using a cutoff of $\log_2(10)$).

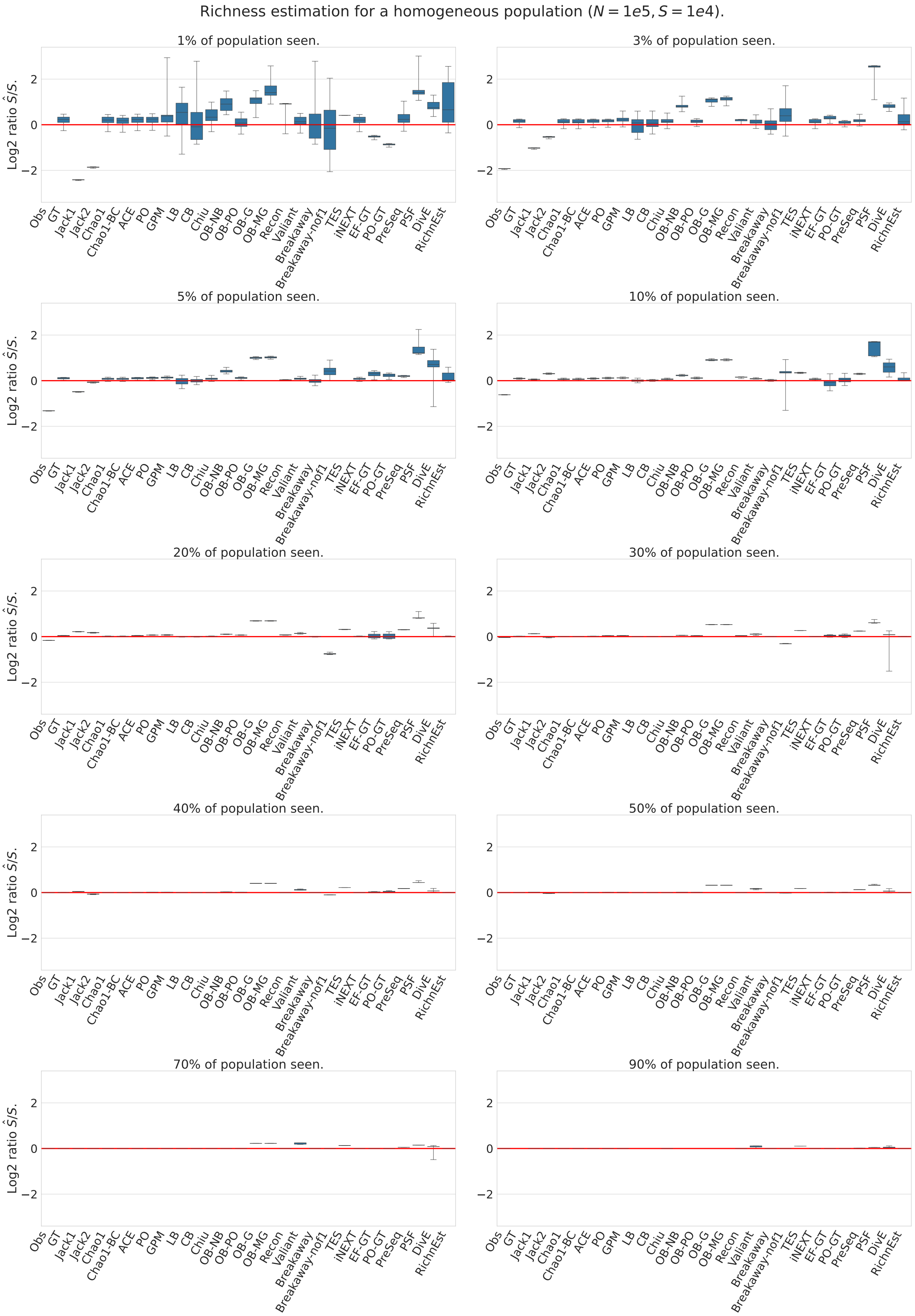

Figure 5: Estimation accuracy for the *homogeneous* population model. Boxplots display the $\log_2$ ratios between the predicted species richness $\hat{S}$ and the true species richness S. If the prediction is correct, $\log_2(\hat{S}/S) = 0$. If $\log_2(\hat{S}/S) > 0$, the estimator overestimates the true richness and if $\log_2(\hat{S}/S) < 0$, the estimator underestimates the true richness. Outliers have been removed (using a cutoff of $\log_2(10)$).

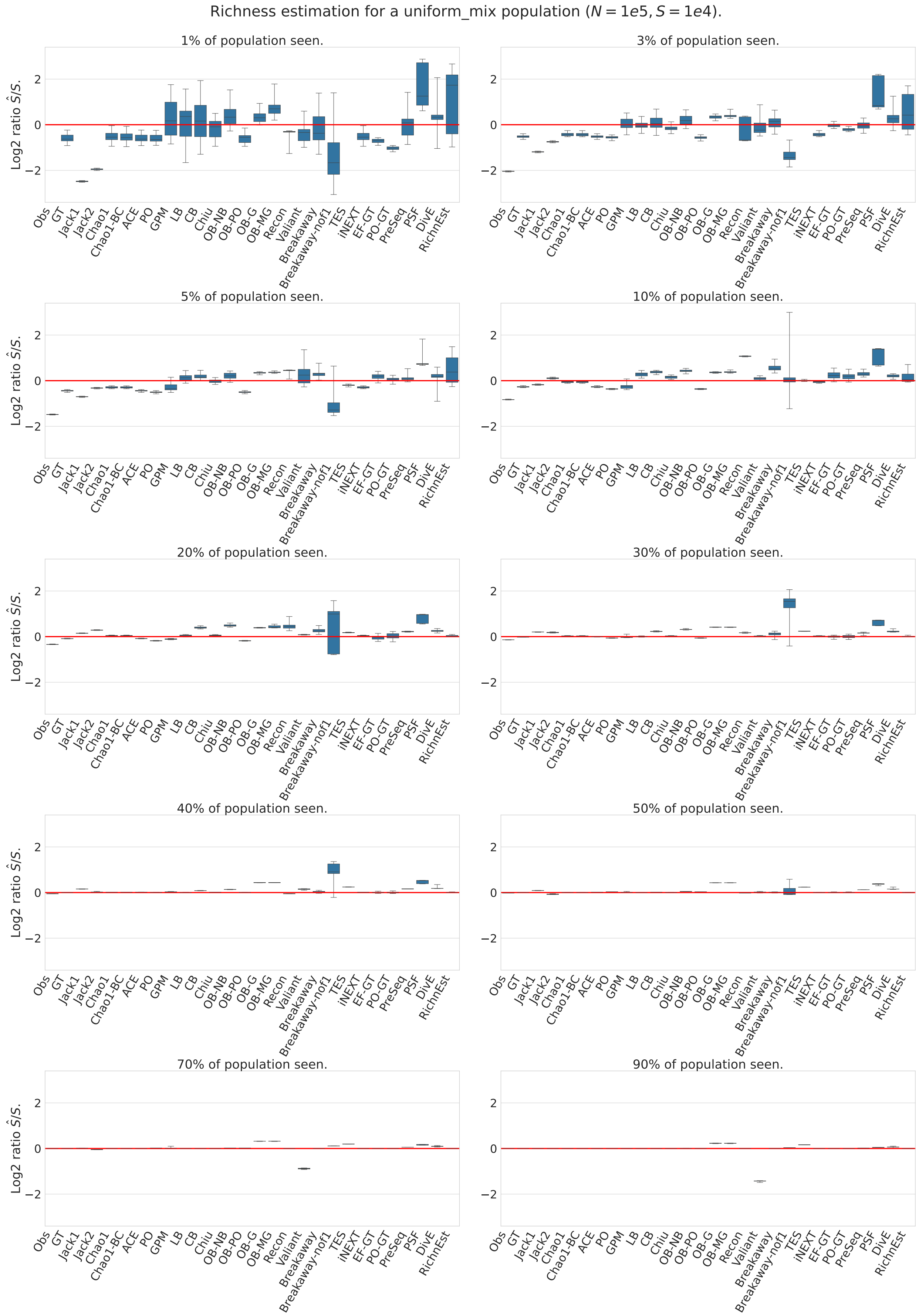

Figure 6: Estimation accuracy for the *uniform mix* population model. Boxplots display the $\log_2$ ratios between the predicted species richness $\hat{S}$ and the true species richness S. If the prediction is correct, $\log_2(\hat{S}/S) = 0$. If $\log_2(\hat{S}/S) > 0$, the estimator overestimates the true richness and if $\log_2(\hat{S}/S) < 0$, the estimator underestimates the true richness. Outliers have been removed (using a cutoff of $\log_2(10)$).

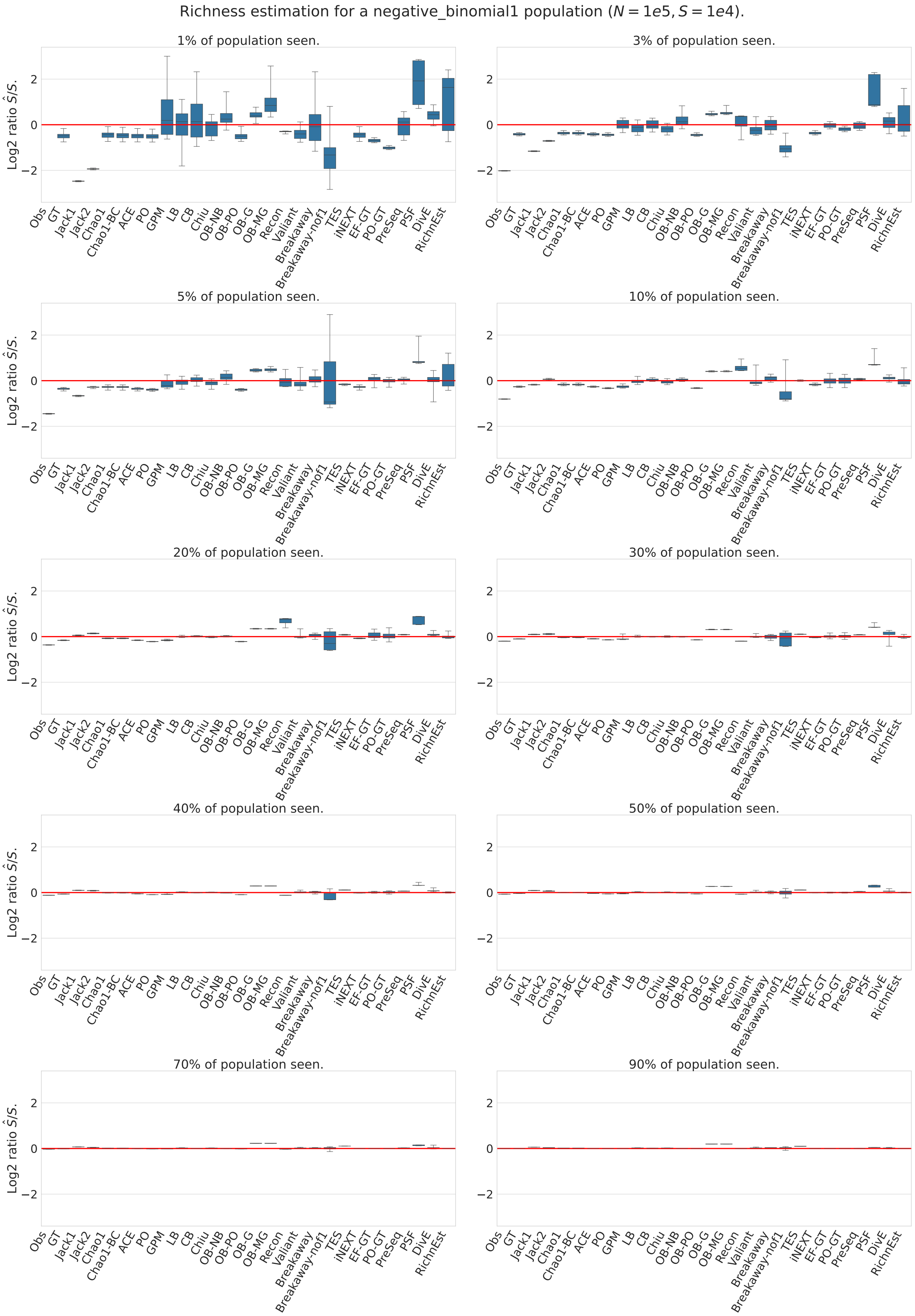

Figure 7: Estimation accuracy for the negative binomial population model ($r = 2, p = 0.02$). Boxplots display the $\log_2$ ratios between the predicted species richness $\hat{S}$ and the true species richness S. If the prediction is correct, $\log_2(\hat{S}/S) = 0$. If $\log_2(\hat{S}/S) > 0$, the estimator overestimates the true richness and if $\log_2(\hat{S}/S) < 0$, the estimator underestimates the true richness. Outliers have been removed (using a cutoff of $\log_2(10)$).

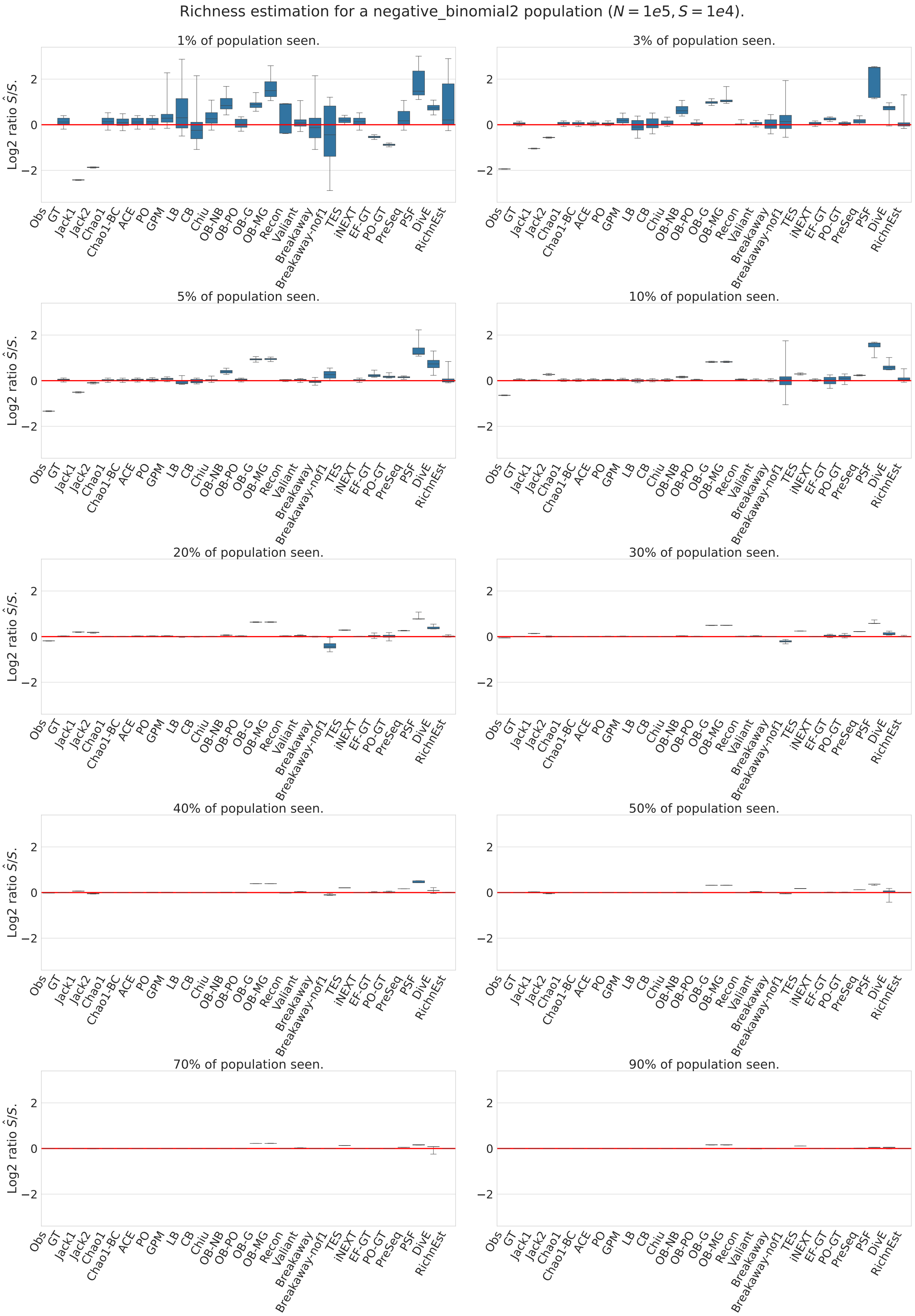

Figure 8: Estimation accuracy for the negative binomial population model ($r = 20, p = 0.2$). Boxplots display the $\log_2$ ratios between the predicted species richness $\hat{S}$ and the true species richness S. If the prediction is correct, $\log_2(\hat{S}/S) = 0$. If $\log_2(\hat{S}/S) > 0$, the estimator overestimates the true richness and if $\log_2(\hat{S}/S) < 0$, the estimator underestimates the true richness. Outliers have been removed (using a cutoff of $\log_2(10)$).

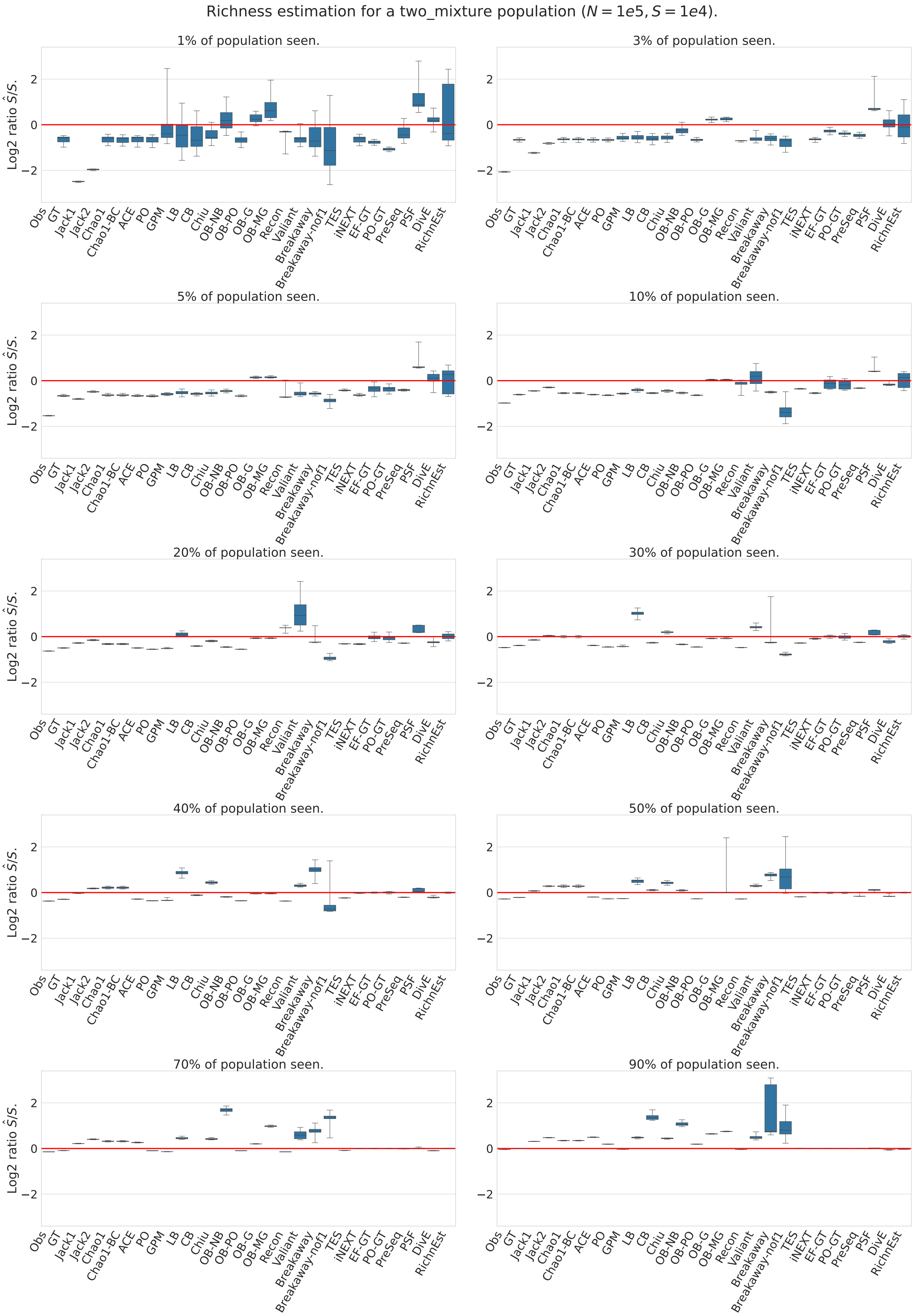

Figure 9: Estimation accuracy for the two mixture population model (mixture of two negative binomial distributions). Boxplots display the $\log_2$ ratios between the predicted species richness $\hat{S}$ and the true species richness S. If the prediction is correct, $\log_2(\hat{S}/S) = 0$. If $\log_2(\hat{S}/S) > 0$, the estimator overestimates the true richness and if $\log_2(\hat{S}/S) < 0$, the estimator underestimates the true richness. Outliers have been removed (using a cutoff of $\log_2(10)$).

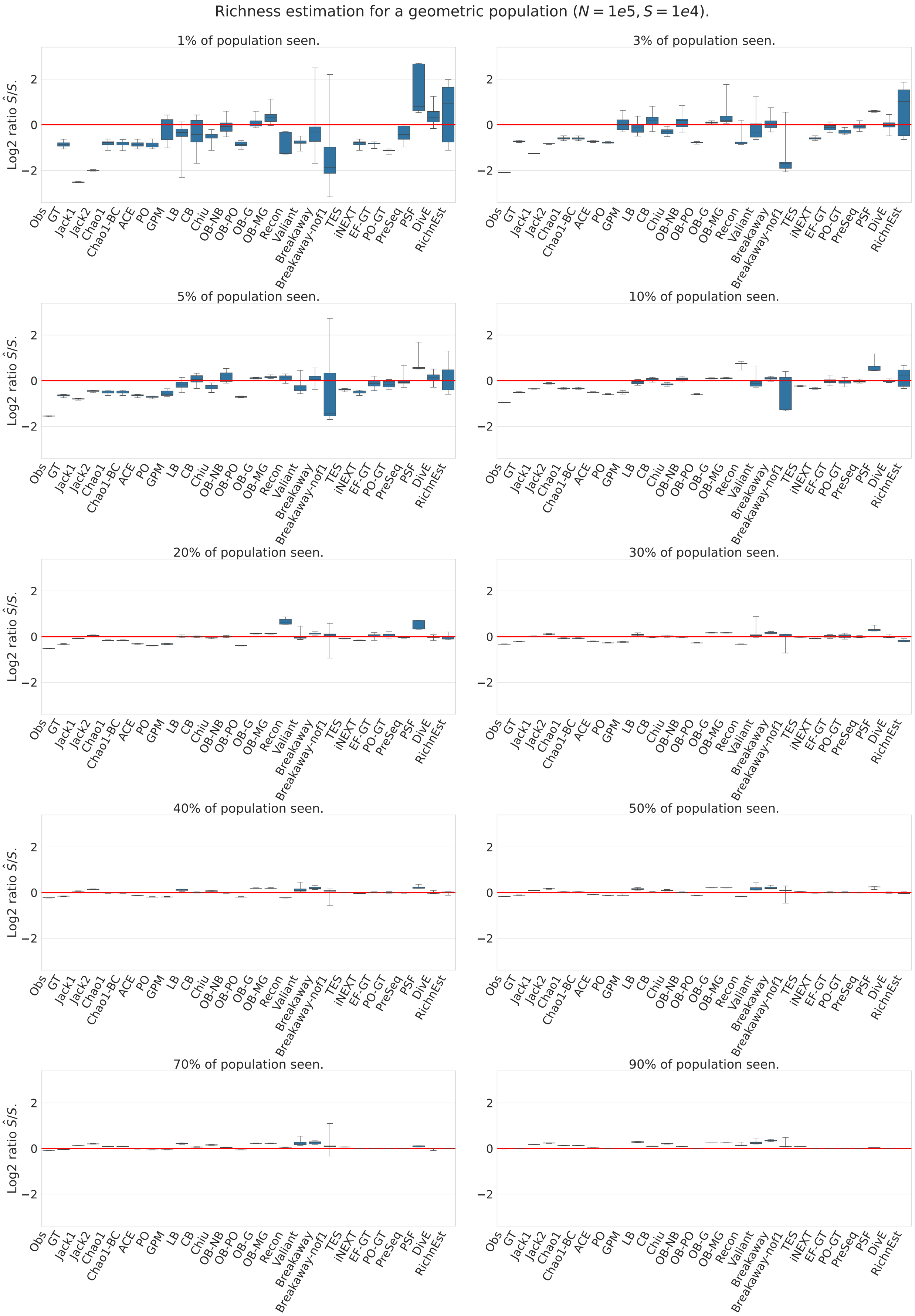

Figure 10: Estimation accuracy for the geometric population model ($p = 1/S$). Boxplots display the $\log_2$ ratios between the predicted species richness $\hat{S}$ and the true species richness S. If the prediction is correct, $\log_2(\hat{S}/S) = 0$. If $\log_2(\hat{S}/S) > 0$, the estimator overestimates the true richness and if $\log_2(\hat{S}/S) < 0$, the estimator underestimates the true richness. Outliers have been removed (using a cutoff of $\log_2(10)$).

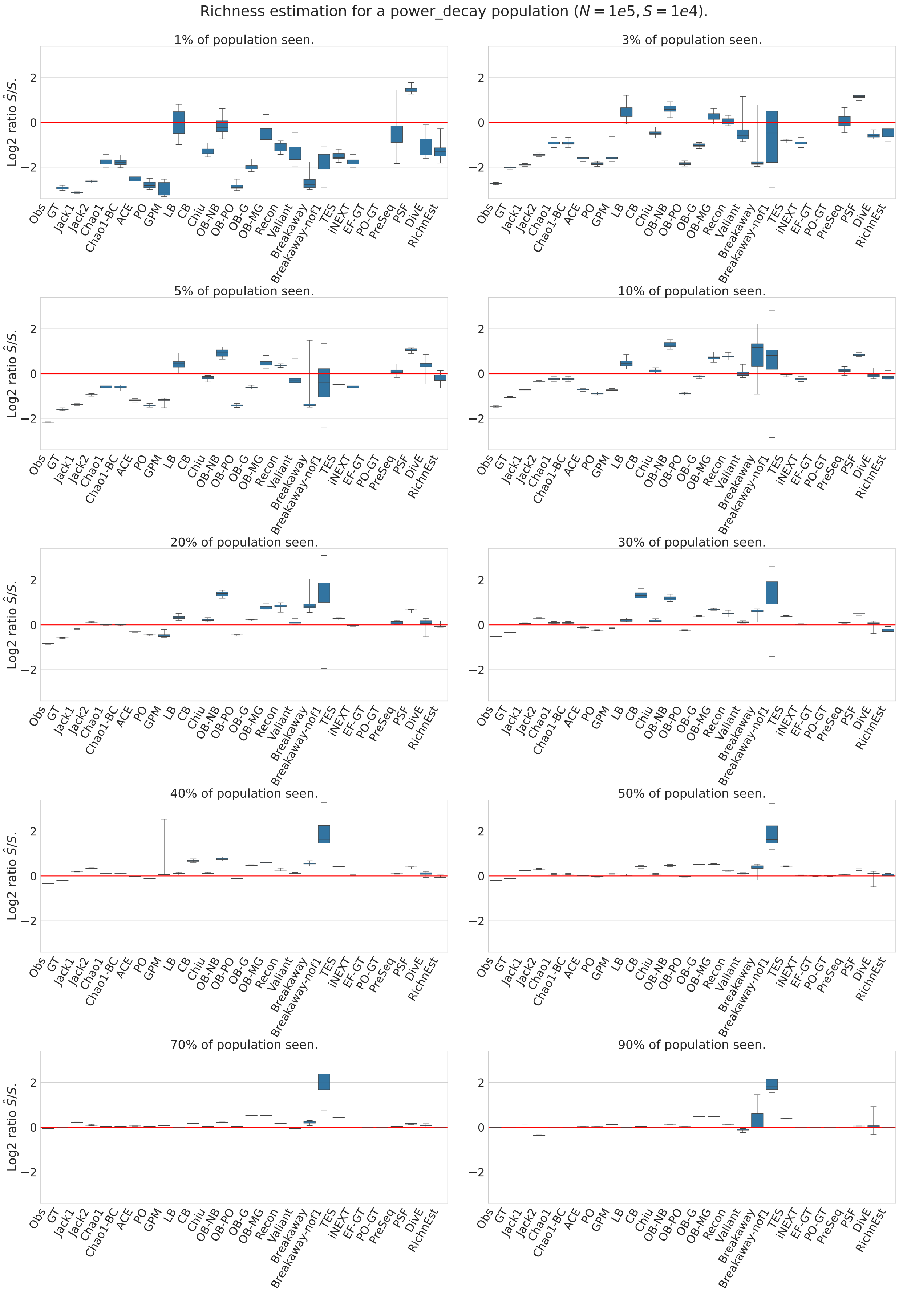

Figure 11: Estimation accuracy for the power decay population model. Boxplots display the $\log_2$ ratios between the predicted species richness $\hat{S}$ and the true species richness S. If the prediction is correct, $\log_2(\hat{S}/S) = 0$. If $\log_2(\hat{S}/S) > 0$, the estimator overestimates the true richness and if $\log_2(\hat{S}/S) < 0$, the estimator underestimates the true richness. Outliers have been removed (using a cutoff of $\log_2(10)$).

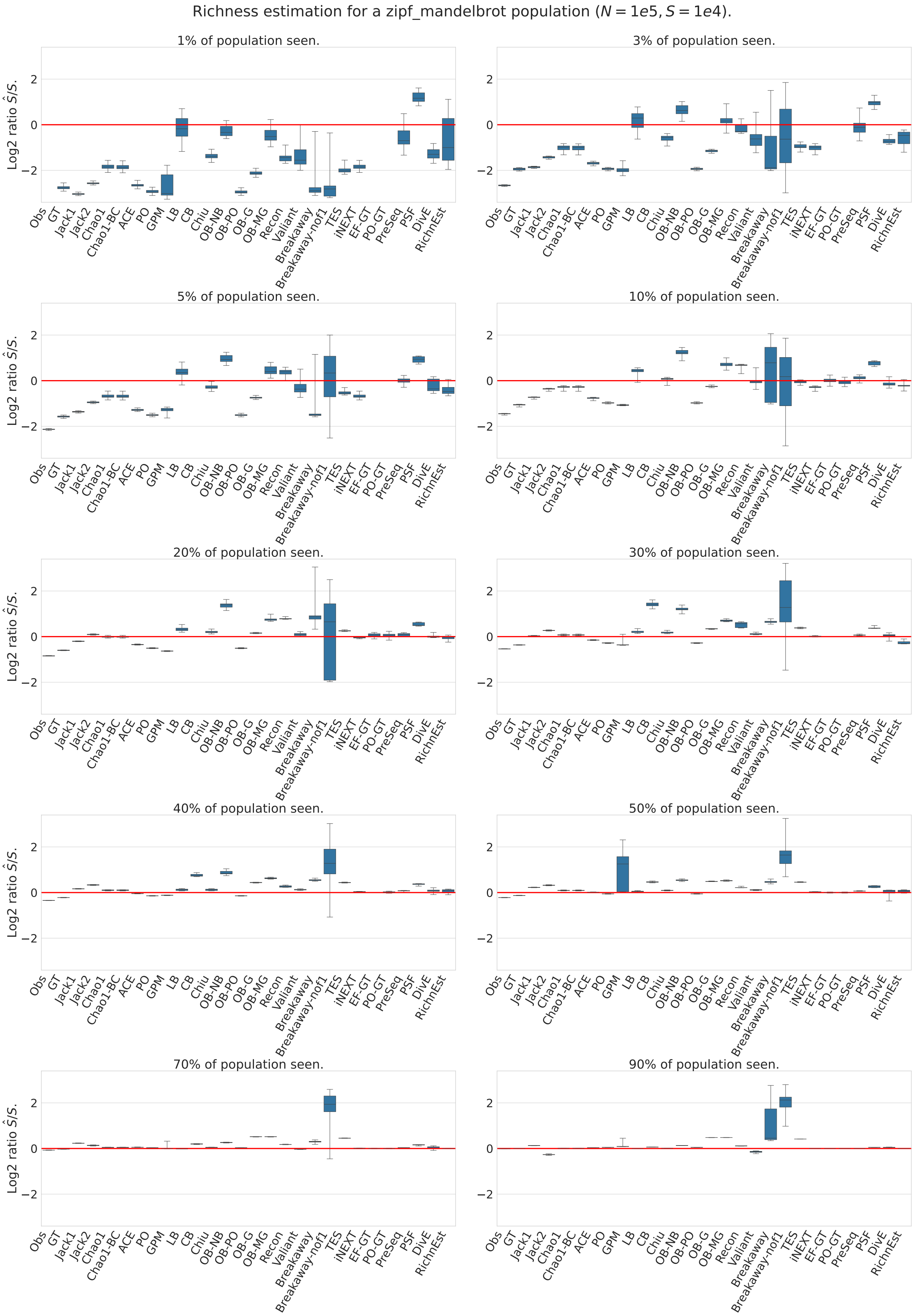

Figure 12: Estimation accuracy for the Zipf-Mandelbrot population model. Boxplots display the $\log_2$ ratios between the predicted species richness $\hat{S}$ and the true species richness S. If the prediction is correct, $\log_2(\hat{S}/S) = 0$. If $\log_2(\hat{S}/S) > 0$, the estimator overestimates the true richness and if $\log_2(\hat{S}/S) < 0$, the estimator underestimates the true richness. Outliers have been removed (using a cutoff of $\log_2(10)$).

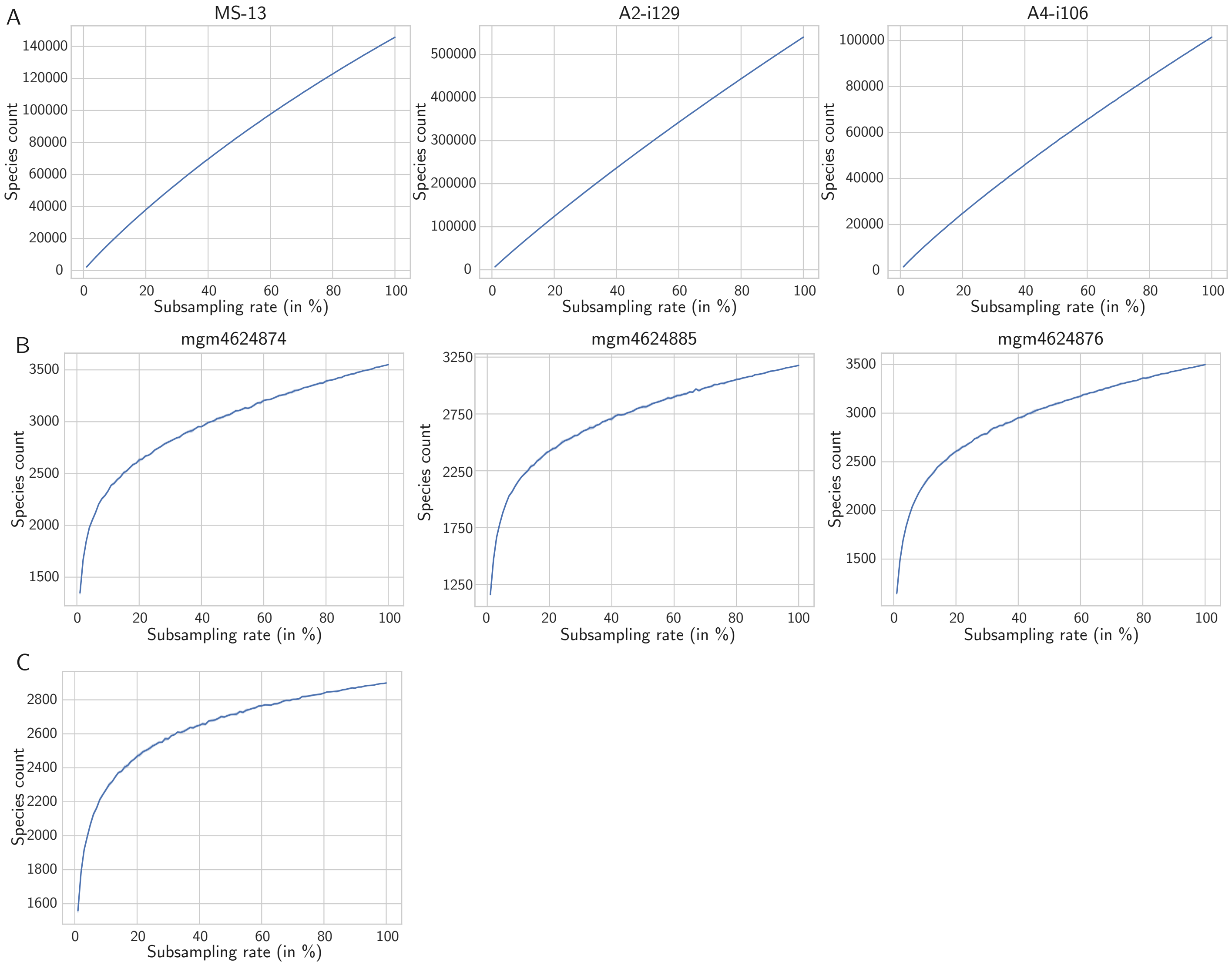

Figure 13: Rarefaction curves for sample richness (number of distinct species in the sample) for different subsampling rates (proportion of the sample included in the subsample), averaged over 100 subsamples. **A** 3 immune repertoire datasets (VDJTools Examples). **B** 3 microbiome datasets (MG-Rast database). **C** Global reef fish data summarized by species (Raw fish data).
